# Nanoengineering‐armed oncolytic viruses drive antitumor response: progress and challenges

**DOI:** 10.1002/mco2.755

**Published:** 2024-10-10

**Authors:** Yan Zhang, Xinyu Shi, Yifan Shen, Xiulin Dong, Ruiqing He, Guo Chen, Yan Zhang, Honghong Tan, Kun Zhang

**Affiliations:** ^1^ Central Laboratory and Department of Medical Ultrasound, Sichuan Academy of Medical Sciences, Sichuan Provincial People's Hospital, School of Medicine University of Electronic Science and Technology of China Chengdu China; ^2^ Department of VIP Clinic General Division, Shanghai East Hospital, School of Medicine Tongji University Shanghai China; ^3^ Department of Medical Ultrasound Renji Hospital Shanghai Jiao Tong University School of Medicine Shanghai China

**Keywords:** nanomaterials, nanotechnology, oncolytic viruses, tumor microenvironment

## Abstract

Oncolytic viruses (OVs) have emerged as a powerful tool in cancer therapy. Characterized with the unique abilities to selectively target and lyse tumor cells, OVs can expedite the induction of cell death, thereby facilitating effective tumor eradication. Nanoengineering‐derived OVs overcome traditional OV therapy limitations by enhancing the stability of viral circulation, and tumor targeting, promising improved clinical safety and efficacy and so on. This review provides a comprehensive analysis of the multifaceted mechanisms through which engineered OVs can suppress tumor progression. It initiates with a concise delineation on the fundamental attributes of existing OVs, followed by the exploration of their mechanisms of the antitumor response. Amid rapid advancements in nanomedicine, this review presents an extensive overview of the latest developments in the synergy between nanomaterials, nanotechnologies, and OVs, highlighting the unique characteristics and properties of the nanomaterials employed and their potential to spur innovation in novel virus design. Additionally, it delves into the current challenges in this emerging field and proposes strategies to overcome these obstacles, aiming to spur innovation in the design and application of next‐generation OVs.

## Graphical abstract

The rapid advancements in nanoengineering have ushered in a plethora of novel nanomaterials and nanotechnologies, invigorating the therapeutic potential of oncolytic viruses (OVs) especially including genetically engineered ones against tumors. Therein, beyond more robust and persistent antitumor response, genetically and chemically engineered OVs are armed with unlimited possibilities such as targeting drug release, combined therapy, magnified immune responses, process monitoring, thus holding high antitumor efficacy for clinical translation.

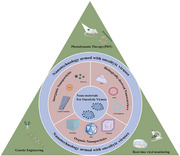

## 1 INTRODUCTION

Viruses present a paradoxical entity in the medical world, serving as a double‐edged sword. While conventional viruses are notorious for their detrimental effects on human health, strategically designed viruses can transform into formidable weapons against cancer. Oncolytic viruses (OVs) were one diamond in the rough, as a biological agent with cancer therapeutic potential. Since the 20th century, the idea of using natural viruses to treat tumors has been put forward.^1^ However, initial efforts were hampered by the strong immune responses and complications associated with natural viruses, leading to underwhelming outcomes and an underestimation of their anticancer potential.^2^ It was not until 1991 that the first genetically engineered OV was reported, heralding a new era for tumor therapy using OVs.^3^

These initial OVs were characterized by their direct and selective cytotoxicity, possessing the capability to preferentially infect and annihilate cancer cells. The antitumor mechanisms induced by OVs are multifaceted, with the principal mechanisms encompassing: (a) the in situ vaccine effect and activation of adaptive immunity through the release of tumor‐associated antigens (TAAs) by lysed tumor cells, thereby aiding immune system recognition and adaptive immune response within the tumor; (b) induction of intrinsic immune responses through the activation of immune cells and cytokine release; and (c) improvement of the tumor microenvironment (TME) by targeting and disrupting the tumor vasculature, altering the dynamics of “hot” and “cold” tumors.^4^, ^5^ In addition, there are some indirect pathways, such as engineered viruses expressing transgenic coding proteins with specific activities can also accomplish the killing of cancer cells.^6^, ^7^ However, the clinical applications of virotherapy encounter several constraints, such as immune clearance of administrated OVs, poor specific tumor targeting, and so on. Thus, promoting the antitumor efficiency of OVs remains a critical focus of ongoing research.

Nanomedicine gives OVs unlimited possibilities. Both nanomaterials and nanotechnologies provide innovative strategies to overcome the limitations of traditional OVs, such as safety concerns, targeting specificity, and replication efficiency.^8^, ^9^ These platforms can be broadly categorized into inorganic/organic nanocomposites,^10^, ^11^ nonmetallic/metal nanoparticles,^12^, ^13^ and biological nanomaterials.^14^ Some pioneering studies have demonstrated that magnetic nanoparticles (MNPs) can augment the infectivity of OVs with antitumor effects through a mechanism mainly due to directed targeting of virus‐nanoparticles and enhanced internalization improvement by the encapsulated capsid coating, leading to enhanced viral progeny formation.^15^, ^16^, ^17^ Additionally, some approaches enhance viral recognition by increasing cellular receptor targeting or modulating TAAs to address a broader range of therapeutic targets.^18^ With the help of nanotechnologies, OVs can achieve more accurate positioning armed with the spatiotemporal controllability. Moreover, biomaterials such as hydrogels can regulate the body's natural immunity to viruses, thereby boosting the persistence and effectiveness of oncolytic immunity, which makes a unique contribution to manage the balance between antiviral and antitumor immune responses.^19^ Furthermore, innovative combinations of nanomaterials and nanotechnologies enable in vivo imaging in tumor diagnostics and facilitate early diagnosis and localization for precisely tumor treatment, garnering increasing attention. Notably, OVs fail to work alone and their synergistic compatibility with various therapeutic modalities underscores the complexity of both TME and OVs themselves.^20^, ^21^, ^22^ The future of OVs thus calls for interdisciplinary collaboration and integrated design efforts across multiple domains to elevate tumor treatment outcomes.^23^

**FIGURE 1:**
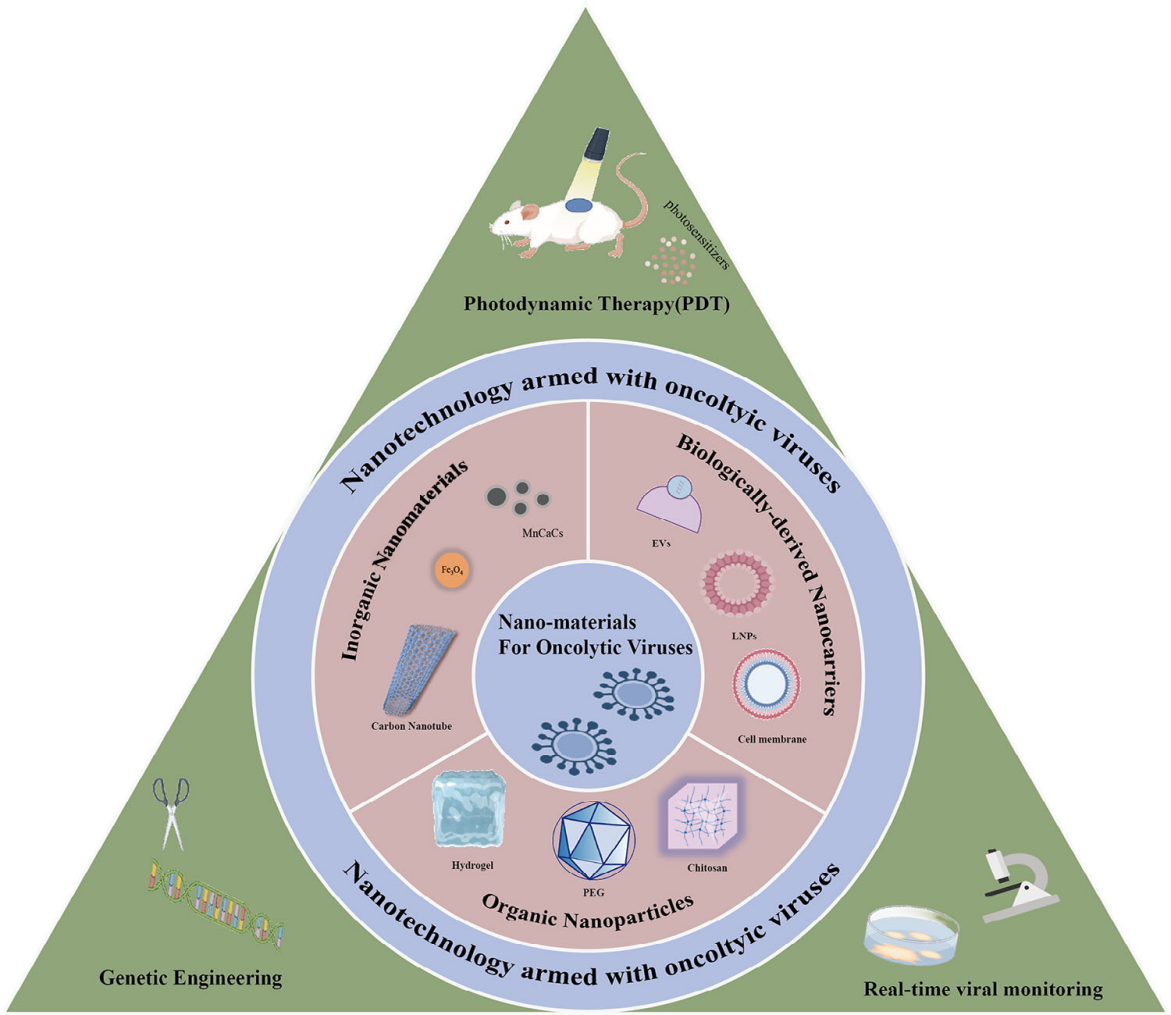
Schematic diagram of nanomaterials and nanotechnologies for OVs by Figdraw. Therein OVs armed with emerging nanomaterials are classified, including inorganic nanoparticles, organic nanoparticles, and biologically derived nanocarriers, along with representative nanotechnologies such as photodynamic therapy, genetic engineering, and real‐time virus monitoring.

This review commences with an overview of common OVs' characteristics, followed by an exploration on their action mechanisms within TME, encompassing aspects of delivery, specific recognition, and immune response. It then delves into how nanomaterials and nanotechnologies can address the limitations of traditional OVs, with a detailed discussion on various categories of nanoplatforms, including inorganic nanomaterials, organic nanoparticles, and biologically derived carriers. The review also covers the incorporation of advanced techniques like photodynamic therapy (PDT), genetic engineering, and real‐time monitoring technologies (Figure 1). Finally, we discuss unresolved challenges, propose promising future directions, and offer perspectives on the clinical translation of OVs, aiming to provide new insights into their design and clinical translation.

## 2 PRECLINICAL AND CLINICAL OVs

### 2.1 Classification and characterization of OVs

There are a wide variety of viruses currently in use in research and clinical settings. Here, we list some of common viruses and their basic characteristics, as well as their advantages and disadvantages, as indicated in Table 1, which is important for selecting appropriate OV.

**TABLE 1:** Common attributes of engineered OVs including types, engineered targets, and so on.

	Genome	Genome size	Natural host	Methods of entry	Blood–brain barrier penetration	Characteristic (advantages or disadvantages)	References
Herpesvirus	dsDNA	150 kb	Humans and other mammals	HVEM, nectin‐1, nectin‐2	–	Large genome (some genes are not necessary for virus replication) to insert large fragments and multiple transgenes	^24^, ^25^
Adenovirus	dsDNA	36 kb	Humans and other mammals	CAR, CD46	–	Ease of genome manipulation; extensive research already	^26^, ^27^
Measles virus	ss(−)RNA	16 kb	Humans	SLAM, CD46	–	Causes fusogenic syncytia formation and cell death; previous natural infection or vaccination cause immunity to the virus	^28^, ^29^
Vaccinia virus	dsDNA	190 kb	Mammals, primarily cattle, and other livestock	Receptor‐mediated endocytosis	–	Rapid replication cycle and high transduction efficiency; replication and transcription of vaccinia virus are exclusively cytoplasmic	^30^, ^31^
Reovirus	dsDNA	123 kb	Humans and other mammals	JAM‐A	+	Maintaining oncolytic capacity in anoxic TME, downregulating the expression of HIF‐1 during infection	^32^, ^33^
Coxsackievirus	ss(+)DNA	28 kb	Humans	CAR/ICAM1/DAF	–	Replicating in the cytoplasm of host cells, reducing the likelihood of insertion mutations	^34^, ^35^
Poliovirus	ss(+)RNA	7.5 kb	Humans	CD155	+	Highly pathogenic in neurons of the human	^36^
Newcastle disease virus	ss(−)DNA	15 kb	Birds, especially poultry (e.g., chickens)	Sialic acid	+	Nonpathogenic in human; replicate up to 10,000 times better in human neoplastically transformed cells than in normal human cells	^37^, ^38^

Currently, several OV products have received approval and are in clinical use, including recombinant human type 5 adenovirus Ankory® for advanced nasopharyngeal cancer exhibiting resistance to conventional radiotherapy or combined radiotherapy and chemotherapy,^39^ talimogene laherparepvec (T‐VEC) for melanoma,^40^ Teserpaturev (G47Δ, DS‐1647) for malignant glioma,^41^ and nadofaragene firadovec‐vncg for nonmuscle‐invasive bladder cancer.^42^ The success of the four OVs provides evidence to support the clinical potential of virotherapy and also paves the way for further research in this field.

### 2.2 Administration method of OVs

The efficacy of OVs is fundamentally tied to their targeted aggregation, which is dictated by their intrinsic properties and the administration method. Moreover, different cellular barriers in organs and tissues, such as the blood–brain barrier or the blood–pancreas barrier, significantly influence the outcomes of administration.

The three predominant administration methods are intratumoral direct injection, intravenous injection, intraperitoneal injection, and intracranial injection. Direct intratumoral injection is particularly beneficial for easily accessible tumors like melanoma, offering controlled OV concentration, direct tumor site anchoring and monitoring, and ease of manipulation.^43^, ^44^, ^45^ This approach simplifies the implementation of treatment regimens and facilitates efficacy comparison. In pediatric retinoblastoma patients, Pascual‐Pasto et al. treated with intravitreous VCN‐01 and observed that the OVs increased potency without causing any inflammatory reactions.^46^ However, due to the limitations of the injection site and the barriers of distant metastasis associated with intratumoral delivery, this method is unsuitable for deep complicated surgical procedures tumors and nonsolid tumors.

In the treatment of cancers with distant target sites, intravenous injection methods can overcome the limitations associated with intratumoral drug delivery. Samson's research indicated that intravenous infusion of oncolytic human orthoreovirus enhanced cytotoxic T cell infiltration and upregulated the PD‐1/PD‐L1 axis in tumors.^47^ However, the systemic immune response induced by intravenous injection necessitates a delicate balance between antitumor activity and potential autoimmune side effects.

Alternative delivery methods include intraperitoneal injection and intracranial injection. Intraperitoneal injection, targeting abdominal organ tumors, ensures high absorption efficiency and simplicity. Low‐dose CF33 intraperitoneal administration was shown to alleviate pancreatic cancer.^48^ Intracranial injection is primarily appropriate for central nervous system tumors. Intracranial herpes simplex virus (HSV)‐1 injection bypasses the blood–brain barrier, enabling direct infection and lysis of tumor cells, thereby stimulating an antitumor immune response.^49^ These methods have limitations as they are only applicable when intratumoral injection and venipuncture are difficult to perform. Currently, they are primarily used on experimental animals.

## 3 BASIC MECHANISMS AND ACTION TARGETS OF OVs‐MEDIATED ANTITUMOR RESPONSES

In recent years, oncolytic virus therapy (OVT) has emerged as an innovative treatment modality that causes tumor lysis through viral infection and replication, as well as stimulates the immune system to target cancer cells, achieving significant progress in clinical practice. The antitumor response triggered by OVs commences at moment of their introduction into the host organism. This comprehensive process encompasses the stages of virus administration, precise targeting and recognition of tumor cells, and the subsequent initiation of cytotoxic effects within TME.

### 3.1 Keys: specific recognition of tumor antigens

**FIGURE 2:**
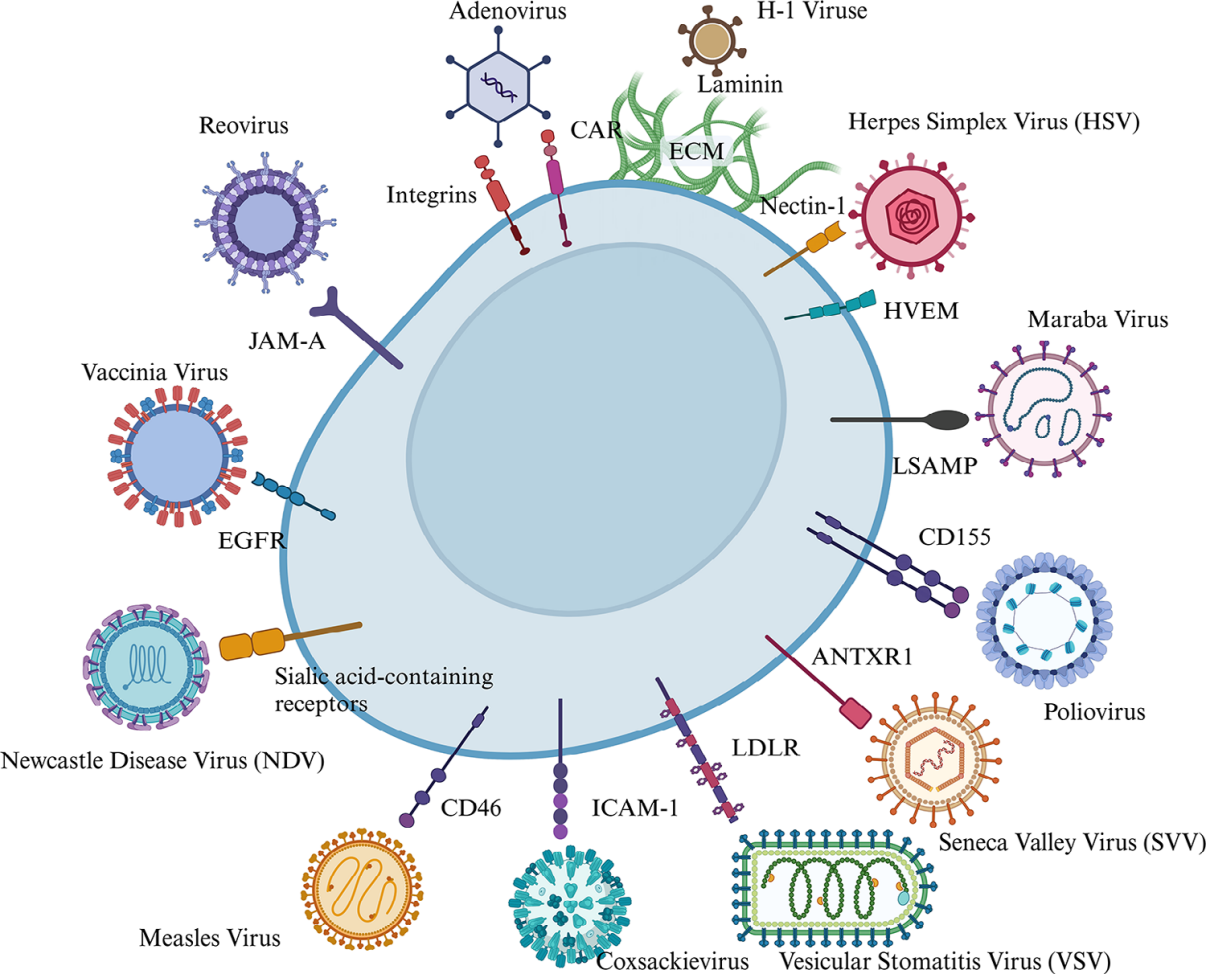
The key to virus recognition of tumor cells, by BioRender. It provides the summary of different OVs and their corresponding molecules for binding with cancer cells.

The initial phase of OV infection critically hinges on specific keys that bind with high affinity to tumor cells. This process is contingent on both the virus's and tumor cells' characteristics, where OVs identify and infect diverse tumor cells via various pathways and targets (Figure 2).^50^, ^51^ First, there are some specific features that naturally exist on the surface of tumor cells, and the OV that matches them happens to target them, thus completing specific recognition and serving as the most crucial key to unlocking the battlefield. CD155, a characteristic marker that is highly overexpressed on tumor cells, but rarely on normal cells, is recognized by Poliovirus (PV) as a receptor.^52^ Thus, PV uses CD155 as an intermediary key to initiate tumor lysis warfare.^53^ Similarly, adenoviruse targeting GP73 is able to infect prostate cancer and exert oncolytic effects.^54^

Another line of thought focuses on the extracellular matrix (ECM), since ECM is usually the barrier for viruses to enter the host cell. Viruses accumulate and reside in ECM in large numbers and then recognize ECM proteins, which in turn trigger viral infection. For example, the sialic acid receptor protein on adherens is vital for the life cycle of oncolytic H‐1 parvovirus, and tumors with high laminin expression show better prognosis after H‐1 virus treatment.^55^

Redesigning antibodies to enhance tumor targeting is another route. Virus‐neutralizing antibodies often greatly diminish virotherapy efficacy. Due to their prevalence, different potencies of antibodies against adenovirus are often produced in human bodies. Niemann's team developed a recombinant antibody that fused adenovirus capsid proteins with single‐chain variable fragments recognizing Polysialic acid (polySia) on tumor surfaces, which effectively targeted related tumors and coincidently overcame neutralizing antibody (nAb) interference.^18^ Similarly, Frederik Wienen's team modified the epidermal growth factor receptor (EGFR) capsid specific for human adenovirus type 5 to efficiently retarget adenoviral vectors to the EGFR in vitro, demonstrated enhanced transduction signals, and provided a mechanism for isolating the organism from adenoviral reduction.^56^

Furthermore, exploiting abnormal pathways in tumor cells offers another avenue for targeted virus infection.^57^ For instance, prostate cancer cells with overactivated RAS signal are more susceptible to HSV‐1 infection because some key viral protein are expressed when transcriptional factors downstream of the RAS pathway are upregulated.^58^

### 3.2 Engaging in the fight: activation of the immune response

The infection of tumor cells by OVs triggers a series of events leading to the replication and spread of the virus within the tumor, culminating in tumor cell lysis. This process releases TAAs, dsRNAs, danger‐associated molecular patterns (DAMPs), viral progeny, and tumor progeny.^59^ The resultant molecules act as signals to activate immune cells, such as antigen‐presenting cells (APCs) including dendritic cells (DCs) and macrophages. These APCs, upon binding to receptors like Toll‐like receptors (TLRs), process and transmit these signals to various immune effector cells. This induction of an immune response through the activation of DCs and adaptive immune cells is called “immunogenic cell death” (ICD).^60^

ICD induced by OVs is a significant phenomenon that extends beyond the virus's lytic effects. Studies have shown that ICD can facilitate the release and repositioning of DAMPs and TAAs, thereby stimulating the immune system. During this process, the released ATP helps recruit APCs. Proteins discharged by cancer cells, such as HSP70, HSP90, and calreticulin, enhance the phagocytic action of DCs on stressed cancer cells. Additionally, cytoplasmic DNA and RNA can activate the secretion of cytokines including type I interferon (IFN‐I) through the cGAS/STING pathway, TLR3 and TLR9.^61^ Collectively, these molecules trigger immune responses, causing inflammation and promoting the activation of antigen‐specific T cells. In the cellular response against OVs, T lymphocytes, especially CD8^+^ T cells, play a crucial role. Their activation hinges on efficient antigen processing and presentation by APCs. Viral infection induces endoplasmic reticulum (ER) stress,^62^ reactive oxygen species (ROS) release,^63^ IFN‐I activation,^59^ and the production of inflammatory cytokines and chemokines, thereby fostering a conducive cellular microenvironment for APCs function. This leads to enhanced OV replication and tumor cell destruction. OVs also promote T cell migration and infiltration by: (a) inducing IFN‐I response and chemokine production; (b) stimulating local inflammatory responses that increase selectins on endothelial cells, facilitating T cell infiltration; and (c) serving as chemoattractants for T cells.^64^, ^65^

Additionally, natural killer (NK) cells play a vital role in antiviral immunity mediated by OVs. By fine‐tuning NK cell functions, OVs can more effectively kill cancer cells and potentially interfere with viral replication within the cancer cells, thereby improving therapeutic outcomes. The significance of carefully regulating NK cell activities in viral vector therapies has been demonstrated in Ncrl knockout mice, which exhibited a more robust response to HSV treatment.^66^

B cells recognized for their significant role in cancer prognosis, get activated in response to OV‐induced TAAs and related markers. Activated B cells first secrete a variety of cytokines, which activate and recruit other effector cells (e.g., T cells) to amplify the killing effect against cancer cells. Moreover, switched memory B cells, capable of evolving into plasma cells, produce antibodies targeting tumors. These antibodies not only promote antitumor responses, but also label tumor cells for destruction by other immune cells. Interestingly, the infiltrated level of memory B cells strongly correlates with the response and prognosis of immune checkpoint blockade treatment. Hence, future OV development might also emphasize B cell‐related activation.^67^, ^68^

TME harbors low‐density populations of immunosuppressive cells, such as regulatory T cells, Th2 cells, tumor‐associated macrophages (TAMs), and myeloid‐derived suppressor cells. These cells secrete immunosuppressive molecules like IL‐10, TGF‐β, and indoleamine 2,3‐dioxygenase (IDO), fostering a “cold” tumor state that impedes DC maturation, antigen presentation, inflammatory cytokine production, immune cell infiltration, and cellular lysis. Hence, targeting these immunosuppressive elements can potentially enhance the efficiency of OVs. For instance, vaccinia virus (VV) encoding a TGFβRII inhibitor gene led to partial regression of tumors in a mouse model.^69^ The combination of the MEK inhibitor trametinib with HSV‐1 enhanced the virus's replication and cytotoxicity in melanoma cell lines.^70^ The presence of OVs can transform this “cold” state into a “hot” proinflammatory phenotype, dramatically altering TME and mitigating tumor immunosuppression. This transformation reinvigorates immune cells in the TME, enhances antigen presentation efficiency, curbs the production of immunosuppressive molecules, and boosts inflammatory responses.^71^, ^72^

Immunosuppressive molecules, such as PD‐L1, CTLA‐4, TIM‐3, BTLA, CD160, LAG3, and 2B4,^73^ not only dampen the tumor's immune response but also impact the efficacy of OVs in infecting tumor cells. Lin's team designed an oncolytic HSV recombinant encoding an aMPD‐1 scFv (OVH‐aMPD‐1), showing that PD‐1 blockade can restore phagocytosis and DCs presentation, significantly inducing ICD in a mouse cancer cell model.^74^ Similarly, a measles virus (MV) expressing a PD‐1 antibody demonstrated improved tumor treatment effects.^75^ Additionally, Ju et al.^76^ combined an OV vector encoding anti‐PD‐L1 with anti‐CTLA‐4 in a mouse tumor model, achieving better tumor suppression than monotherapy. Therefore, designing OV vectors to downregulate PD‐L1 and CTLA‐4 presents promising prospects in inducing and supporting sustained antitumor immunity.^76^

**FIGURE 3:**
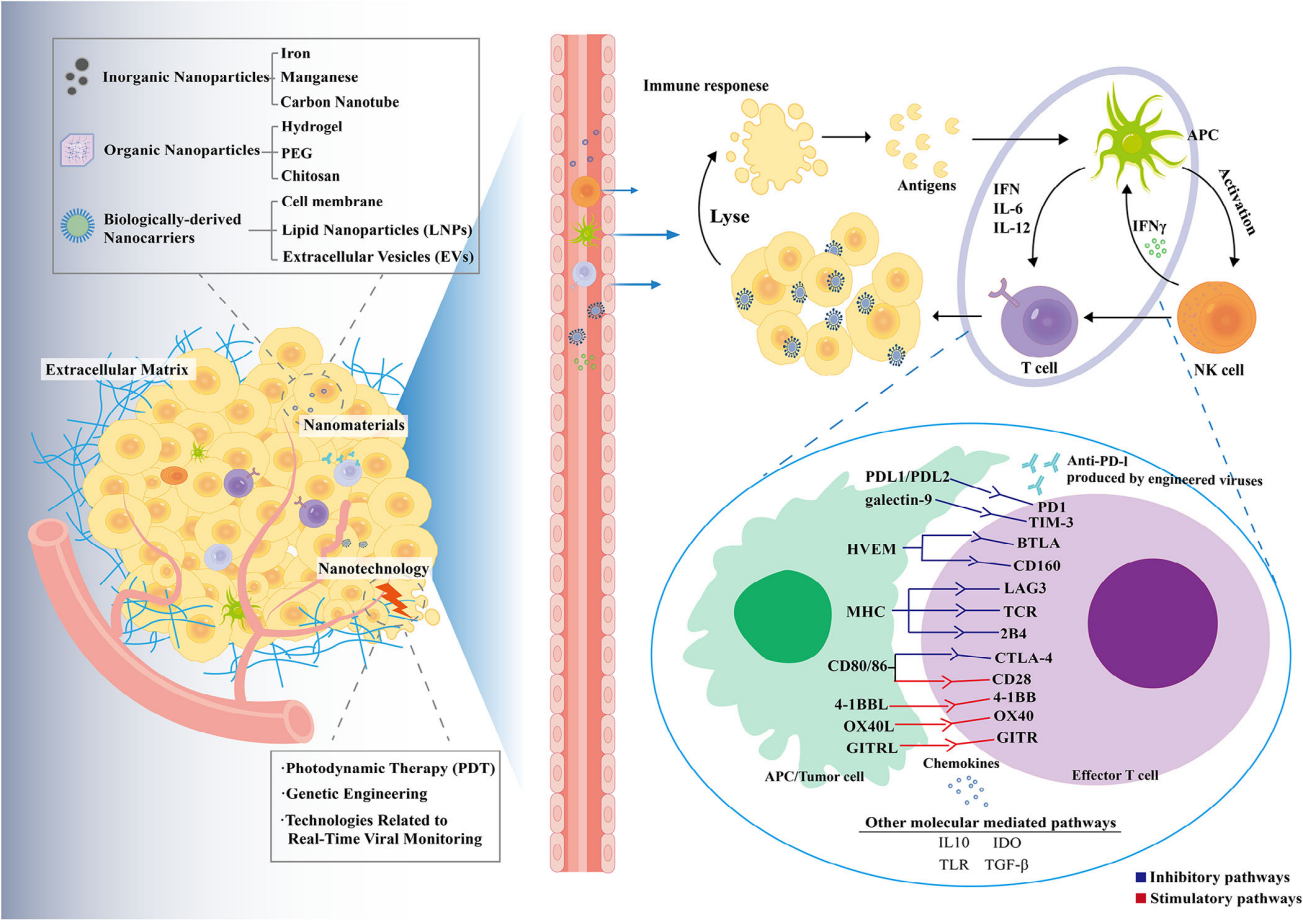
Generalized overview of oncolytic immunogenicity of OVs armed with nanotechnology. Following local or systemic administration of OVs, nanotechnologies/material‐armed OVs enter the tumor microenvironment through various routes, gradually exposing themselves to the host's antiviral immune responses. Those OVs exhibit advantages in targeted delivery to tumor tissues during sustained administration, thereby enhancing their transmission efficiency to tumor tissues. Subsequently, viral infection targets tumor cells, leading to viral dissemination throughout the tumor tissue, resulting in tumor cell lysis and release of viral/tumor antigens. This event attracts and activates innate and adaptive antiviral and antitumor immune cells, leading to the activation of diverse molecular pathways that may result in widespread tumor cell lysis and tumor regression.

In summary, the immune response induced by OVs constitutes an intricate organism. It is evident that various immune cells contribute synergistically to OVT. This synergy is achieved by recognizing, attacking, and eradicating tumor cells, thereby significantly enhancing the therapeutic efficacy. Such a multifaceted immune engagement firmly establishes OVT as a promising and evolving strategy in the realm of cancer treatment. Figure 3 illustrates the series of immune responses triggered after OVs enter the body. Nanomaterials and related technologies can serve as carriers to enhance the therapeutic effects of OVs, as described in the following sections.

### 3.3 Battlefield: TME remodeling

TME encompasses both the internal and external milieu of tumor cells, comprising not only the tumor and stromal cells but also noncellular components like ECM, tumor vasculature, and various secretory factors (e.g., cytokines, chemokines, growth factors, and proteases).^77^ Characterized by low tumor antigen levels and the presence of immunosuppressive cells and signaling molecules, TME is often described as an immunologically “cold” environment. OVs, as a potent class of immunotherapies, not only target and lyse tumor cells specifically but also reshape the TME, fostering an environment favorable for the influx and activation of antitumor immune cells. This transformation creates an immune “hot” environment, conducive to long‐term tumor‐specific immunity. The mechanisms through which OVs remodel the TME include vasculature normalization, ECM decomposition, and metabolic reprogramming reversal.

#### 3.3.1 Vascular system normalization

Research indicates that rapid tumor progression consumes substantial oxygen, rendering the TME hypoxic. This hypoxia triggers an overproduction of angiogenic factors like vascular endothelial growth factor (VEGF) during tumorigenesis, leading to vascular abnormalities that are not conducive to T cell proliferation, invasion, and activation, and becomes a major obstacle to antitumor immunity.^78^ Furthermore, many proangiogenic factors, including VEGF, possess immunosuppressive properties. Tumor‐associated endothelial cells also contribute to T cell inactivation by expressing PD‐L1.^79^

Therefore, OVs are designed to target tumor angiogenesis pathways and reverse tumor immunosuppression. Many OVs possess intrinsic antivascular properties. For instance, adenoviruses encode the E1A gene, which inhibits VEGF expression in cancer cells. Saito et al.^80^ discovered that an oncolytic adenovirus expressing a mutant E300A, lacking p1 binding sites, significantly reduced its ability to inhibit VEGF expression in pancreatic cancer cells (PCCs), leading to increased tumor vascular density. Oncolytic VV also exhibits antiangiogenic properties and VEGF levels in the tumor significantly diminished after viral infection.^81^ Breitbach et al.^82^ utilized about 1000 continuous histological sections to create a three‐dimensional reconstruction of subcutaneous colon cancer tumors, revealing how intravenously injected vesicular stomatitis virus directly infects and damages the tumor vascular system within 24 h. HSV has been shown to directly infect the tumor endothelium in vivo and significantly reduce the mean vascular density of ovarian cancer.^83^

In addition, many studies of OVs have also attempted to target angiogenic factors through gene modification. Typically, novel oncolytic adenoviruses expressing FP3 (RdB/FP3) has been shown to significantly reduce VEGF expression levels and vascular density and increase the apoptosis of tumor endothelial cells and tumor cells, confirming the effective inhibition of RdB/FP3 on VEGF‐mediated tumor angiogenesis in vivo.^84^ Coinfection of replication‐deficient adenoviruses (Ad‐Flk1‐Fc) expressing soluble vascular EGFR with replication‐capable “helper” viruses significantly inhibited the progression of human prostate tumors and reduced CD31 staining in mice.^85^ Gene‐modified OVs targeting angiogenic factors have shown promise in reducing VEGF levels and vascular density, thus inhibiting tumor angiogenesis. However, there is a possibility of vascular perfusion resumption following viral clearance. Therefore, combining OVs with antivascular drugs could further enhance therapeutic efficacy by increasing endothelial permeability and viral delivery.

#### 3.3.2 ECM decomposition

Studies have shown that CAFs can be activated by tumor‐derived molecules such as TGF‐β and IL‐6, inducing excessive deposition of ECM proteins (mainly collagen) and producing a dense and tough matrix with elevated interstitial fluid pressure, thus forming a strong physical barrier against intratumoral invasion by immune cells.^86^ A typical example of ECM affecting immune invasion is pancreatic ductal carcinoma (PDAC). The high‐density, fibrotic tumor stroma constitutes up to 80% of the tumor mass and plays a crucial role in PDAC's invasion, drug resistance, immunosuppression, and poor prognosis.^77^

OVs are genetically modified to express hyaluronidase, which leads to the disintegration of ECM and promotes infection and immune cell infiltration of OVs. Tedcastle and his colleagues have cloned actin‐resistant DNase (DNase I) and hyaluronidase (rhPH20) into conditionally replicating Group B adenoviruses that express ECM‐degrading enzymes, which enhance the therapeutic efficacy of xenografts of colorectal adenocarcinoma.^87^ Oncolytic adenoviruses expressing PH20 hyaluronidase showed destruction of hyaluronic acid (HA) in ECM, promoting the spread of OVs.^88^ OVs can also target tumor–matrix interactions. In vitro studies have shown that the infection with LOAd713, a trimerized membrane‐bound isoleucine zipper (TMZ)‐CD40L‐armed oncolytic adenoviruses that activate myeloid cells and scFv to block IL‐6 signaling, significantly reduces related factors to dampen ECM synthesis, such as TGF‐β1, type I collagen, and fibroblast growth factor 5.^89^ This is based on the mechanism by which excessive production of IL‐6 by TAM leads to the increase of TGF‐β and thus induces fibroplasia.

#### 3.3.3 Metabolic reprogramming reversal

The metabolic state within TME is a key determinant of the antitumor immune efficacy of OVs. A major impediment is the metabolic exhaustion of immune cells. Thus, OVs can rejuvenate immune cell vitality by targeting specific metabolic pathways. As a paradigm, genetically engineered oncolytic VV expressing the metabolic regulatory factor leptin (VV‐leptin) can improve mitochondrial function and quality by activating T cell AMPK signaling, thereby reversing the VV‐induced T cell mitochondrial dysfunction itself.^90^ Inhibiting glycolysis by promoting oxidative phosphorylation with dichloroacetate can downregulate IDO to improve antitumor immune response to oncolytic Newcastle disease virus (NDV).^91^

Glycolytic metabolism appears to play a different role in responding to the viral life cycle. In the initial stages, increased aerobic glycolysis within TME provides sufficient energy and building blocks for viral replication. However, in the later stages, excessive glycolysis leads to negative feedback to defend against viral replication and spread. Therefore, the effect of metabolic reprogramming on antitumor immune response is more fully considered in the design of optimal OVs.

### 3.4 Cell death pathways induced by OVs

Cell pyroptosis is a soluble and inflammatory programmed cell death pathway distinct from apoptosis, which has attracted widespread attention in tumor immunotherapy in recent years.^92^ Studies have found that OVs infection of tumor cells can induce the activation of Gasdermin proteins (such as GSDME). Gasdermin proteins are a class of pore‐forming proteins that can form pores on the cell membrane, leading to cell swelling, rupture, and content release, which is known as cell pyroptosis. Jing Lin and colleagues found that oncolytic parapoxvirus can activate antitumor immune responses by inducing cell pyroptosis mediated by GSDME. The virus inhibits the ubiquitination degradation of GSDME protein, boosts the intracellular GSDME level, induces GSDME cleavage activation, and promotes cell pyroptosis.^93^ Zhang et al.^94^ validated that Coxsackievirus B3 (CVB3) could induce colon cancer cells to undergo pyroptosis by activating caspase‐3, leading to GSDME cleavage.

The OVs‐induced cell death not only directly kills tumor cells, but also reshapes TME through activating immune responses. This process converts originally “cold” tumors insensitive to immune therapy into “hot” tumors sensitive to immune therapy, thereby enhancing the effectiveness of immune treatment. For instance, a novel treatment nanoprodrug combines ROS/pH dual‐responsive signal transduction and transcription factor 3 inhibitor with oncolytic HSV type 1. After HSV‐1/nanoprodrug complexes demonstrate high tumor penetration property and significantly induce gasdermin‐E (GSDME)‐mediated cell death.^95^ Additionally, Wang et al.^96^ designed a novel therapy combining a dual‐responsive DNA methyltransferase inhibitor nanoprodrug with oncolytic HSV. The inhibitor nanoprodrug releases the epigenetic inhibitor 5‐azacytidine, which upregulates the expression of GSDME at the gene level; meanwhile, oncolytic HSV further enhances the intracellular levels of GSDME by reducing ubiquitination and degradation of GSDME. The combined use of both demonstrates a synergistic enhancement of GSDME‐mediated cell death. In summary, the strategy of the combination therapy inducing tumor cell death represents a novel, promising approach and may pave the way for personalized tumor treatment.^96^

## 4 EMERGING STRATEGIES FOR ENHANCING OVs‐BASED ANTITUMOR EFFICIENCY

After reviewing numerous studies on enhancing the efficiency of OVs, we have briefly summarized four key approaches to improve curative effect: (1) increasing viral transduction and replication rates, (2) strengthening virus selectivity for tumor cells, (3) combining OVs with other drugs and therapeutic modalities, and (4) suppressing cellular antiviral immunity. Meanwhile, smart platforms constructed by highly modifiable nanomaterials serve as multifunctional and powerful weapons, which could potentially enhance the efficiency of OVs to infect tumor cells and elicit antitumor immune responses through the four approaches mentioned above.

### 4.1 Increasing viral transduction and replication rates

In addition to an active targeting navigation, enhancing the efficiency of viral transduction and replication is also essential for developing superior OVs’ formulations. Gene modification of the replication‐related genes (e.g., the P gene of MV virus) may affect viral replication rates. By replacing the P gene of the MV vaccine strain with that of the wild‐type MV (IC‐B), it is possible to modify the replication dynamics of the engineered virus and promote its spread throughout the tumor.^97^ Another viable strategy involves the application of OVs in conjunction with specific compounds. SMCs, namely second mitochondria‐derived activator of caspases mimetic compounds, have demonstrated the ability to facilitate the replication of M1 OVs.^98^ It has been reported that the replication of HSV‐1 in melanoma cells is increased when treated with trametinib.^70^ Certain malignancies display aberrations in the IFN signaling pathway, making these cancer cells more vulnerable to viral infections. In such cases, employing IFN response inhibitors, such as TPCA‐I (an IKK‐2 inhibitor) or Ruxolitinib (a JAK1/2 inhibitor), can augment the efficiency of oncolytic viral infections.^99^ However, whether this strategy works effectively depends on more in‐depth research on chemicals that assist viral replication, as well as advancements in encapsulation and delivery technologies for the combination of OVs with anticancer drugs. This is a point at which nano‐based carriers could potentially come into play, given their significant impact on loading multiple therapeutic agents, enhancing delivery efficiency and expanding bioavailability. Catalyzing the oxidation of lactate to pyruvate and H_2_O_2_, lactate oxidase (LOX) has the potential to overcome the limitation on transduction efficiency of adeno‐associated virus serotype 2 (AAV2) imposed by the pH‐dependent protease.^10^ Tseng et al.^100^ encapsulated AAV2 and LOX with HA, and observed that viral infection increased in parallel with the level of LOX.

Additionally, utilizing nanodelivery systems, such as liposomes^101^ and nanohydrogels,^102^ can effectively overcome biological barriers, protecting OVs from degradation and ensuring their successful intracellular delivery. Previous reports have pointed out that nanocarriers also contribute to stable encapsulation and increased accumulation of OVs in solid tumors. The efficacious concentrations of oncolytic adenoviruses loaded by a delivery system consisting of liposome particles and Escherichia coli reached 170 times higher than bare OAs.^103^ Moreover, the transfer of OVs from infected cells to uninfected cells could be promoted by incorporating extracellular vesicles (EVs) into the viral systems.^104^ These approaches all have the potential to enhance the efficiency of OVs’ delivery and replication.^105^

### 4.2 Enhancing virus selectivity to tumor cells

Tumor‐specific replication refers to the high replication rate and activity of OVs in tumor cells, while their replication in normal cells is limited. This property makes OV a potential tumor treatment tool, as it can selectively infect and kill cancer cells without causing too much damage to normal tissue. Some OVs have been engineered through different strategies to make sure that they can replicate specifically in tumor cells while minimally infecting normal cells. Common strategies primarily involve modifying genetic loci and protein expression via bioengineering technology. For one thing, some genes crucial for inhibiting both cancer and viral replication in normal cells, may be inactivated or missing in tumor cells. By deleting the elements on the OVs that interact with those genes, high targeting of tumor tissues can be achieved. For example, p53, a tumor suppressor that promotes cell cycle arrest and terminates the viral replicative lifecycle in host cells,^106^ could be inactivated by the EIB element in adenoviruses through ubiquitination. The replication ability of H101, a recombinant replication type 5 adenovirus lacking E1B, is weakened in normal cells while still maintained in tumor cells.^107^ Furthermore, the ICP34.5 protein that is responsible for blocking autophagy and facilitating viral replication is modified by deleting two gammas to attenuate replication in normal cells.^108^

For another, viral replication selectivity may be enhanced when viral binding to its natural receptors is ablated and a new specificity domain that targets entry into tumor cells is added.^109^For example, due to the wide distribution and lack of tumor specificity of the natural receptors CD46(5,6) and SLAM(7‐9) of attenuated live MV, Nakamura et al.^110^ developed a pseudoreceptor system that allows viruses retargeted to tumor‐selective CD38, EGFR or EGFR mutant vIII (EGFRvIII) to efficiently enter cells through their respective targeted receptors in vitro and in vivo, but not through CD46 and SLAM. It is obvious that the targeted viruses demonstrated specific receptor‐mediated antitumor activity.

In addition, incorporating nanoengineering technology represents an alternative strategy to improve the specificity of tumor targeting. In some studies, the intrinsic tumor‐targeting property of certain nanomaterials (e.g., graphene oxide [GO],^111^ neural stem cells [NSCs]/glioblastoma [GBM]‐derived nanostructured membranes^112^) exhibiting is leveraged to optimize the delivery and distribution of OVs in vivo. Previous studies have highlighted that external intervention including ultrasound, microbubbles, hyperthermia,^113^ additional field and others, facilitate greater penetration of nanoparticles into tumor. Under the influence of the external magnetic field, MNPs such as Fe_3_O_4_ nanoparticles,^12^, ^114^ also navigate OVs to solid tumor cells via a technology known as magnetic navigation.

There are also compounds that exhibit tumor‐targeted effects in response to specific environmental stimuli and are employed in the development of OV systems through nanoencapsulation, conjugation with nanocarriers, and so on. An illustrative example is the LOX containing nanoparticles designed by Pan‐Chyr Yang's group,^10^ which was endowed with high reactivity of lactate in the TME and provide active guidance for endogenous infection of OVs. The primary mechanism for tumor‐selective delivery of the nanoengineering‐armed OVs described above may be ascribed to the navigating compounds themselves, so to speak. However, considering the enhanced permeability and retention effect associated with nanocarriers,^113^ these nanoplatforms not only serve as delivery vehicles but also serve as secondary targeting effects. More detailed illustrations will be provided in Section 3 and there is still much room for exploration of potential nanoplatforms in this scenario.

### 4.3 Combined therapy with other therapeutic agents and modalities

The synergistic combination of OVs with other therapeutic agents as well as antitumor therapeutic modalities can significantly enhance oncolytic outcomes. For instance, CDK4/6 inhibitors have been shown to deplete retinoblastoma protein levels, leading to more efficient viral replication and increased production of OVs in carcinomatous fetuses. This results in an effective antitumor response, as evidenced in mouse xenograft sarcoma models.^115^ The alkylating chemotherapeutic drug TMZ is another example that combined therapy would improve the treatment outcomes as TMZ has been found to increase oncolytic adenovirus replication and activity in mouse lung and breast cancer cells.^116^

Sun et al. explored the combination of OVs with bacterial therapies by conjugating liposome‐encapsulated OAs onto Escherichia coli BL21 and the E. coli‐lipo‐OA therapy bridged by liposomes significantly enhanced the antitumor immunity.^103^ Additionally, tumor‐fighting modalities including chemodynamic therapy and PDT exert synergistic killing effect when OVs are combined with nanomaterials such as Fe_3_O_4_ nanoparticles,^117^, ^118^ Mn‐based nanoparticles,^119^ carbon‐based materials,^120^ and so on. The combined therapy mediated by OVs and nanoplatforms will be explained and demonstrated in detail in Section 4.1 through examples. Other types of nanomaterials also demonstrate potential in combating cancer, for example, chitosan (Cs)^121^, ^122^ and EVs^123^ have been reported to activate the immune system. Furthermore, nanoplatforms not only provide safe and versatile carriers for Ovs, which enable diverse complementary therapies to improve antitumor effect, but also facilitate the loading of additional therapeutic agents with tumor‐suppressive property,^95^ allowing for the effective integration of multidimensional therapies within the nanometer scale. Therefore, the diverse advantages of nanomaterials possessed in tumor destruction ranging from promoting tumor cell lysis to modulation of TME and mobilization of immune cells should also be taken into consideration when developing nanoengineering‐armed OVs with improved efficiency.

### 4.4 Suppressing cellular antiviral immunity

The virus will induce the antiviral immune response of the body after affecting cells, leading to the short of virus cell entry, cell fusion and target cell killing ability. By inhibiting the neutralization reaction of antibody, the transfection efficiency of virus can be improved. MVs are rapidly inactivated by antimeasles nAbs, which limits their clinical performance as oncolytic agents. Bah et al.^124^ disrupted the natural receptor interaction by replacing it with a homologous gene and generated a fully retargeted MV that was resistant to neutralization in measles immune human serum. Compared with control MV, recombinant MV binding CDV F and H glycoproteins remained fully infectious when exposed to high concentrations of mixed measles immune human serum.^124^ In addition, recent literature has shown that OVs provide a barrier against enzymatic or immune system degradation through complex delivery of cationic polymers, and are also capable of cellular uptake by OVs. Chen et al.^125^ synthesized a methionine and N‐(3‐aminoprolil) methacrylamide modified acrylamide cationic block polymer for the delivery of OVs. They demonstrated that the OVs delivery by electrostatic complexation of NAD with OV can improve transfection efficiency in the presence of nAbs.^125^ Liposomes,^126^ nanohydrogel,^102^ lipid nanoparticles (LNPs),^127^ extracellular vehicles,^102^, ^128^ and other nanomaterials also have been reported to be capable of providing protection to OVs from immune neutralization and boosting the efficacy of systemic oncolytic adenovirus administration, which will also be demonstrated in the following section.

Overall, the triumph of OVs hinges on judicious selection of viruses, effective administration, identifying tumor‐specific traits, mobilization of tumor immunity and active remodeling of TME, providing clues to strategies that maximize the clinical potential of OVs. Meanwhile, the combination of OVs and nanomaterials is emerging as a promising approach to implementation of these strategies. Enhanced therapeutic response endowed by the intrinsic properties of nanomaterials, as suggested by a growing number of researches,^129^ is shedding new light into enhancing viral targeting and infection of cancer cells while safeguarding healthy tissue.

## 5 NANOMATERIALS‐ARMED OVs

While we have extensively discussed the in vivo antitumor mechanisms of OVs (Figure 3), their clinical efficacy encounters challenges from systemic and local barriers within TME. For patients with tumors associated with severe tissue infiltration, the occurrence of peripheral organ metastases, and treatment‐resistant tumors, higher doses and higher efficiencies of OV are required for tumor clearance.^130^ Also, viral capsids alone are readily recognized as pathogens by the innate immune system and cause immune elimination upon systemic administration of OVs. At the same time, conventional OVs may also be poorly enriched due to tumor metastasis as well as barrier effects,^131^ and even intravenous OVs may cause serious side effects such as cytokine release syndrome.^132^

With advancements in biotechnology, particularly nanomaterials, the treatment with OVs is no longer confined to using the virus alone. Nanoparticles classified as inorganic, organic, and biologically derived nanocarriers confer special mechanical properties through their unique internal structures and properties, stabilize encapsulated virus particles, improve transmembrane transport, neutralize antibodies against OVs as well as activating tumor immunity.^133^ This section will delve into the distinct roles of these novel technologies and materials in therapeutics, aiming to provide insights into the development of future treatments.

### 5.1 Inorganic nanoparticles

#### 5.1.1 Iron

Iron nanoparticles, with diameters ranging from 1 to 100 nm are minute particles typically enveloped in shells composed of high‐molecular‐weight polymers, silicon, or hydroxyapatite. Presently, the more widely employed iron nanoparticles are generally synthesized from superparamagnetic or ferromagnetic Fe_3_O_4_ or γ‐Fe_2_O_3_. Under the action of an applied magnetic field, directional motion can be achieved to facilitate positioning and separation from the medium.^134^ Simultaneously, the active groups coupled on the shell layer can be functionalized by combining with a variety of biomolecules, such as proteins, enzymes, antigens, and antibodies. Iron nanoparticles combine the properties of magnetic and polymer particles, offering biocompatibility, biodegradability, and biomedical functionality.

**FIGURE 4:**
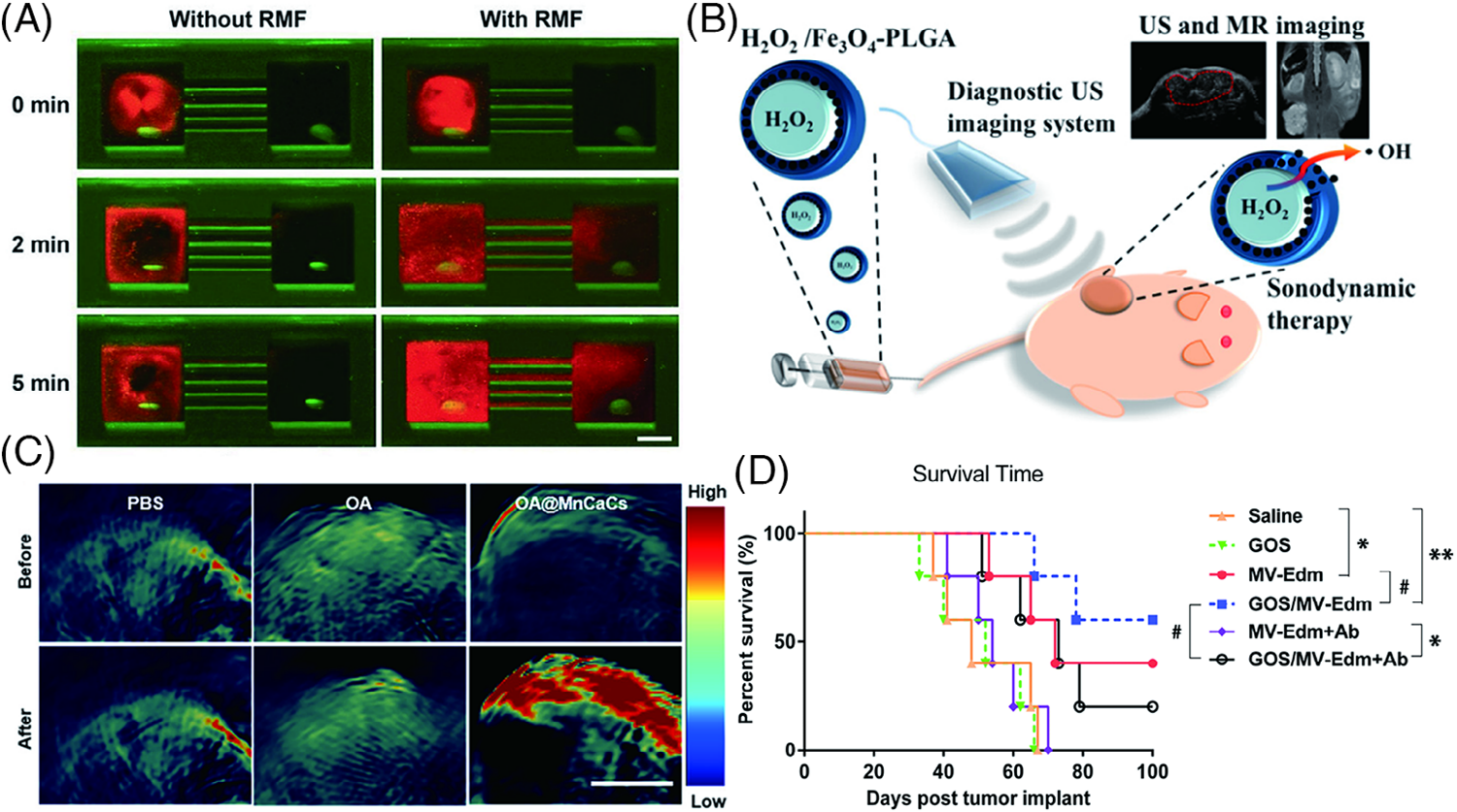
Inorganic nanoparticles applied to arm OVs. (A) Fluorescence imaging of the microfluidic device: this series of images showcases the response of Cy3‐labeled cell robots to an applied rotating magnetic field (RMF) of 10.3 mT at 17 Hz over varying durations (0, 2, and 5 min). Scale bar: 4 mm. The images vividly visualize the movement of the cell robots under magnetic influence, highlighting their migration toward cancer cells. Reproduced from Ref. 12. Copyright © 2022 Wiley‐VCH GmbH. (B) Schematic diagram of H_2_O_2_/Fe_3_O_4_–PLGA (PLGA: poly(lactic‐co‐glycolic acid)). When exposed to ultrasound, Fe_3_O_4_ undergoes a Fenton reaction with H_2_O_2_ and provides the capability for MR imaging due to the unique properties of Fe_3_O_4_. Reproduced from Ref. 137. Copyright © 2016, American Chemical Society. (C) PA imaging of HbO_2_ (oxyhemoglobin) in 4T1‐tumor‐bearing mice: intravenous administration of OA@MnCaCs, the dual‐functional approach not only facilitates the imaging of the physiological state of the tumor but also signifies the therapeutic impact of the MnCaCs‐biomineralized OV on altering TME toward a more oxygenated and therapeutically responsive state. Scale bar: 3 mm. Reproduced from Ref. 119. Copyright © 2019, American Chemical Society. (D) Survival plot of a nude mouse model of HeLa human cervical cancer tumors: in the presence of MV‐neutralizing serum antibodies, there was a significant survival benefit in the Hela mouse model receiving GOS/MV‐EDM compared with MV‐EDM (black and purple curves). Reproduced from Ref. 11. Copyright © 2019, Mao Xia et al.

It has been demonstrated by current evidence that iron nanoparticles can cooperate well with OVs, displaying their unique value in terms of production efficiency, targeted delivery, contrast imaging, and tumor killing. Iron nanoparticles show significant potential in OVs‐targeted therapy by prolonging virus–tumor interactions and shielding viral surface receptors from immune clearance. Both the work on polyethyleneimine‐coated Fe_3_O_4_‐NPs with silica^15^ and Howard's team using nanomagnets from magnetotropic bacteria coloaded with HSV1716 demonstrate that these materials can be magnetically controlled to extend tumor site retention and shield the virus from nAbs, enhancing immune cell recruitment and improving tumor cell lysis efficiency.^64^ Peptide‐modified cell robots coated with Fe_3_O_4_‐NPs enable targeted delivery of OVs through magnetically controlled rolling and migration in microchannels. This composite exhibits enhanced driving force for adhesion and penetration into deep tumor tissues, thereby achieving effective tumor‐targeting immunotherapy under controllable directional propulsion^12^, ^114^, ^135^ (Figure 4A). Due to the presence of Fe_3_O_4_, this material also provided additional MR imaging capabilities^136^, ^137^ (Figure 4B). Meanwhile, Fe_3_O_4_‐NPs can induce oxidative stress, activate iron death, and inhibit the proliferation and invasion of ovarian cancer.^138^ Magnetic Fe_3_O_4_‐NPs generate heat under an alternating magnetic field and target the tumor site to kill the tumor, achieving an effect similar to photothermal therapy and PDT.^117^, ^118^ In addition, Fe_3_O_4_‐NPs are the classical reagents for realizing the Fenton reaction by coencapsulating Fe_3_O_4_ with H_2_O_2_, in which liquid H_2_O_2_ can be easily encapsulated by the hydrophilic core of polymer body. Then, this nanomaterial was injected into the circulation of mice, and under ultrasound, the H_2_O_2_ encapsulated in the core was released and reacted with Fe_3_O_4_ in a Fenton reaction (Formula 1), forming a large amount of therapeutic ROS, thereby helping OVs to induce oxidative damage. ROS produced by oxidative stress promote nonimmune responses in tumors by altering TME, cytokine profiles, and APCs.

(1) $$\begin{array}{c} Fe^{2+}+H_{2}O_{2}\to Fe^{3+}+HO^{\cdot }+OH^{-}\\ Fe^{3+}+H_{2}O_{2}\to Fe^{2+}+HO^{2\cdot }+H^{+} \end{array}$$

#### 5.1.2 Manganese

Mn‐based nanoparticles have demonstrated a wide range of application scenarios in tumor immunotherapy,^139^ potentially elucidating why Mn‐based nanoengineered OVs function well in antitumor activity: (A) manganese nanoparticles, due to their inherent structure of inactivity, are widely used as a biocompatible nanocarriers for delivering immunotherapeutic agents including OVs to improve their bioavailability^140^, ^141^; (B) manganese nanoparticles participate in the regulation of TME and promotes antitumor immunity through synergy with OVs. Specifically, OVs armed with manganese oxide nanoparticles can release Mn^2+^ through reduction reaction, which can generate ROS through Fenton/Fenton‐like reactions with endogenous H_2_O_2_. As a result, the destruction of oxidative stress of biomolecules, such as proteins, lipids and nucleic acids in tumor cells and cell death induced by ROS would be also observed while OVs are exerting antitumor effect.^142^ At the same time, Mn^2+^ strongly promotes the immune response through the proliferation of cytotoxic T lymphocytes and the maturation of DCs.^143^ (C) Manganese nanoparticles demonstrate potential for adding imaging‐related functionality to OVs, as Mn‐based contrast agents are the most common class of TME‐sensitive magnetic resonance nanocontrast agents.^144^ In weakly acidic TME, they can release Mn^2+^ with five unpaired electrons and increase the probability of contact between the paramagnetic centers of Mn^2+^ and water molecules, which can significantly shorten the longitudinal relaxation time of water protons and achieve the longitudinal relaxation time of magnetic resonance imaging (MRI)‐T1 enhancement.^145^

A novel bimetallic ion‐based biological coating composed of copper and manganese has been developed for oncolytic adenoviruses. This mineral‐coated virus can evade immune surveillance while the two metal ions serve to reduce the expression of PD‐L1 in tumor cells and promote the release of immune‐related factors. This dual action converts “cold” tumors into “hot” tumors, thereby enhancing the immune response against the tumor.^146^

Huang et al.^119^ coated adenovirus with calcium and manganese carbonate (MnCaCs) to form bio‐mineralized nanoparticles that can easily dissolve in the acidic TME, release Mn^2+^, and can convert endogenous H_2_O_2_ into oxygen (O_2_) to enhance the replication ability of OA, thus significantly improving the antitumor efficacy. Meanwhile, Mn^2+^ and O_2_ enable T1 modality MRI and photoacoustic imaging, which can provide real‐time tumor imaging (Formula 2). This method accomplished the dual tasks including tumor lysis and enhanced contrast imaging, demonstrating the great potential of Mn nanoparticles^119^ (Figure 4C). Notably, there is currently limited research on how to achieve the multifunctional capabilities of Mn‐based nanoengineered Ovs, which may enable both tumor ablation and cancer imaging. However, the findings reported by Zhao et al.,^147^ Zou et al.,^148^ and Sun et al.^149^ regarding successful cancer imaging using Mn‐based nanoplatform have provided a theoretical basis for the feasibility of developing Mn‐based nanoengineered OVs capable of MRI.^147^

(2) $$\mathrm{Mn}O_{2}+H_{2}O_{2}+2H^{+}\;\to Mn^{2+}+2H_{2}O+O_{2}$$

#### 5.1.3 Carbon‐based materials

Due to their unique physical and chemical properties, carbon‐based materials, including carbon nanotubes, graphene, GO, and some composites, offer promising avenues in tumor therapy for drug delivery,^13^ bioimaging,^150^ tumor immunity,^11^ photodynamic, and photothermal therapies.^120^ Many nanoparticles can also be constructed as complexes with graphene to provide even more unique properties. For instance, the combination of iron oxide and graphene has both magnetic and optical properties, enabling targeted drug delivery and remote drug release.^151^ Its high near‐infrared (NIR) absorption, large specific surface area, and abundant functional groups can be used for effective biomolecular loading or biocoupling.^152^ Sun's group prepared ultrasmall graphene oxide nanocomposites (NGO) with lateral dimensions less than 10 nm and covalently grafted polyethylene glycol (PEG) star polymers. The results demonstrate photoluminescence spanning from visible light to NIR wavelengths, suitable for cellular imaging in low‐background environments. In addition, the aromatic anticancer drug adriamycin was loaded onto the NGO sheet with high capacity by simple physical adsorption. The antibody‐conjugated NGO selectively delivers drugs to cancer cells and achieves controlled release under the acidic TME and acidic intracellular conditions within lysosomes, thereby selectively killing the cancer cells.^153^ What is more, Yue et al. found in vitro experiments that GO is capable of multiple functions, such as antigen uptake, transport, and autophagy‐mediated antigen presentation in bone marrow dendritic cells (BMDCs).^154^ Dextran‐functionalized reduced graphene oxide (rGO‐dextran) demonstrated enhanced antigen delivery and presentation, as well as the promotion of inflammatory cytokine release. In a B16 melanoma mouse model, it exhibited a higher ratio of CD4^+^ to CD8^+^ cells and smaller tumor volumes, indicating that GO serves as a promising nanocarrier of vaccines.^111^ With fabricating a polyethyleneimine–GO nanosheets–PEG–folic acid (PEI–GONSs–PEG–FA) composite, this study successfully encapsulated the attenuated MV (MV‐Edm), forming the GONSs/MV‐Edm complexes. In the HeLa mouse tumor model, this composite markedly enhanced the targeted delivery and therapeutic efficacy of OV while effectively overcoming the impact of nAbs on viral treatment, thereby significantly improving mouse survival. These results demonstrate the tremendous potential of graphene in augmenting OVT efficacy, offering a promising avenue to more effective cancer treatment^11^ (Figure 4D).

### 5.2 Organic nanoparticles

Polymers are synthesized through techniques like emulsification and precipitation, using natural and synthetic materials. This versatility allows for varied structures, enabling effective drug encapsulation or conjugation. Such diversity aids in creating specific structures for loading various drugs, including hydrophilic, hydrophobic substances, various molecules, proteins, and nucleic acids. Their multifunctionality enables controlled drug release and targeted immunological responses.

#### 5.2.1 Hydrogel‐related polymer nanoparticles

Hydrogels comprise hydrophilic polymer chains forming a three‐dimensional network that enables them to swell and absorb water while maintaining structural integrity^155^. In aqueous environments, hydrogel surfaces become moist and pliable, resembling human body conditions and exhibiting excellent biocompatibility.

**FIGURE 5:**
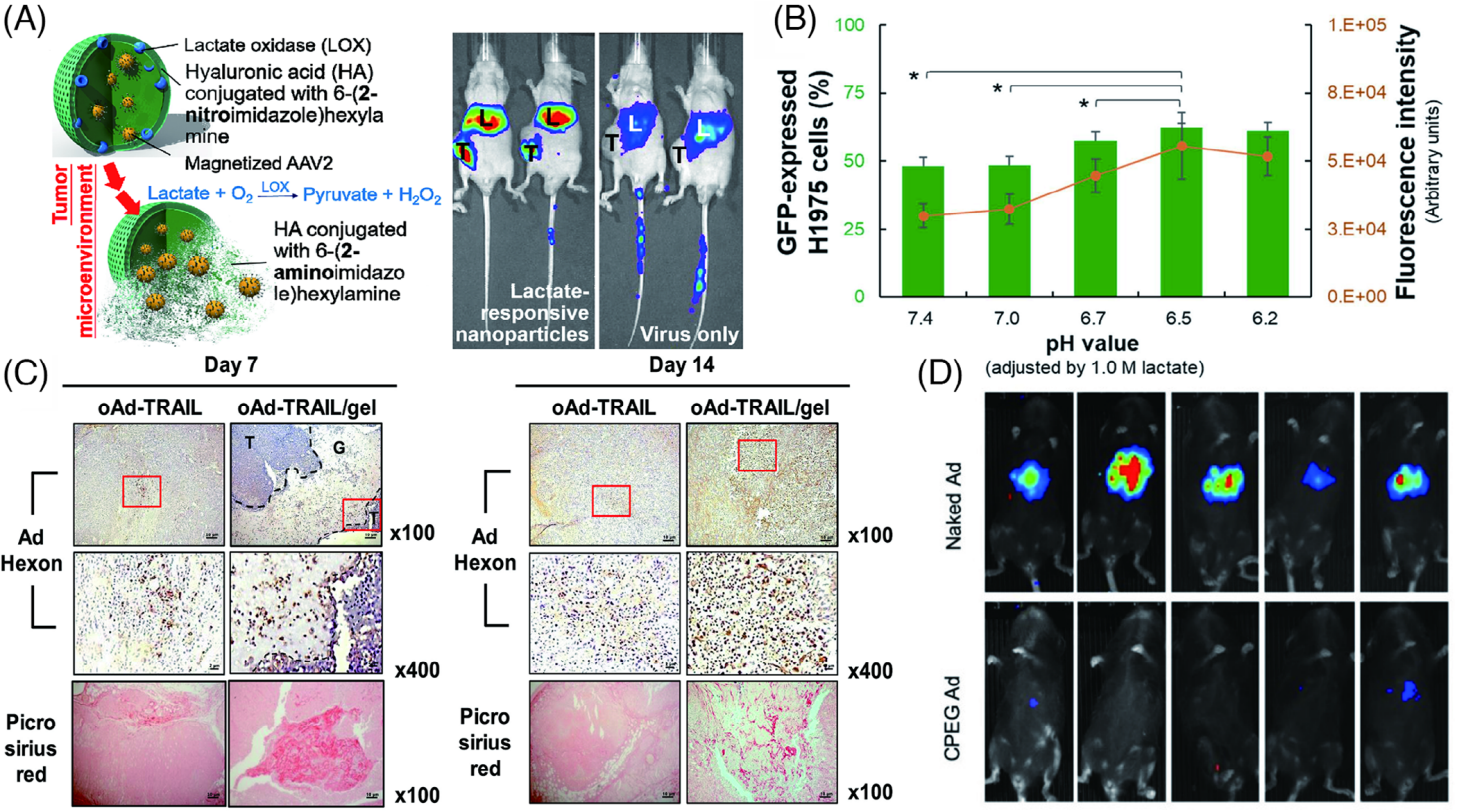
Organic nanoparticles applied to arm OVs. (A) Schematic illustration of a carrier consisting of hyaluronic acid (HA) coupled to 6‐(2‐nitroimidazolyl) hexylamine to “magnetize” the local release of recombinant AAV2. The carrier contains LOX, which oxidizes lactate in TME, creating a bioreductive environment within the carrier that converts hydrophobic 2‐nitroimidazole to hydrophilic, and electrostatically dissociates the carrier and payload. Reproduced from Ref. 100. Copyright © 2018, American Chemical Society. (B) Flow cytometry was performed to determine the percentage and fluorescence intensity of GFP‐positive H1975 cells after 6 days of transduction with AAV2 alone, at varying pH values adjusted by lactate. The results indicated that acidic conditions not only led to an increased number of cells expressing GFP but also resulted in an increased amount of GFP expression by the cells. Reproduced from Ref. 10. Copyright © 2022 S.‐Ja Tseng et al. Published by Elsevier Ltd. (C) Immunohistochemical analysis of HaP‐T1 tumor sections at 7 and 14 days: Ad hexon staining detected virus in tumor tissue, while picrosirius red staining detected collagen hydrogel. A comparison revealed that, under the influence of Ad/collagen hydrogel system, the virus exhibited enhanced persistence within the tissue. Original magnification: ×100 and ×400. The scale bar represents 10 and 2 µm for ×100 and ×400, respectively. T, tumor region; G, gel region. Reproduced from Ref. 162. Copyright © 2017 Elsevier Ltd. (D) Bioluminescence imaging in mice: the liver of mice injected with CPEGAd shows reduced transduction of the administered virus and decreased liver isolation. Reproduced from Ref. 168. Copyright © 2020, Brugada‐Vilà et al.

Hydrogels show delicate responsiveness to bodily stimuli, being sensitive to external stimuli such as pH,^156^ temperature,^157^ electric fields and magnetic fields,^158^ light,^159^ and biological molecules such as glucose and enzymes.^160^ Hydrogels can respond to these stimuli by undergoing volume expansion or contraction, thereby fulfilling their functions, such as drug release.^161^ And this sensitivity allows tailoring hydrogels to specific conditions using the body's diverse environments. Typically, in lactate‐rich TME, LOX‐loaded hydrogel nanoparticles dissociate the carrier and virus, targeting infection at the site^100^ (Figure 5A). Additionally, LOX transforms the high lactate environment into pyruvate, dissolving the outer polymer shell and lowering the local pH, enhancing the protease activity of the adenovirus AAV2 capsid for improved viral transfection^10^ (Figure 5B).

With emergence as a cornerstone in targeted therapy, hydrogels are adeptly used for the delivery and controlled release of OVs. Leading this innovation, Jung's team engineered a biocompatible and biodegradable gelatin hydrogel platform designed to express the tumor necrosis factor (TNF)‐related apoptosis‐inducing ligand OV, that is, adenoviruses,^162^ marking a paradigm shift in targeted cancer therapy (Figure 5C). Similarly, the Gtn‐HPA hydrogel, containing oncolytic CRAd and CIK cells, is formulated for sustained delivery of IL‐12 and IL‐15, targeting tumor tissues precisely.^163^ Advancing innovation, another team developed microporous GelMA hydrogel (CellDex) capsules, encapsulating engineered cells like Thc, including tumor‐specific therapeutic proteins and OVs. The in vivo performance of these Celldex capsules with ThCs shows exceptional longevity, viability, and the ability to target tumor cells effectively. Also, the advancements in ex vivo primary brain tumor models demonstrate this approach's therapeutic impact.^164^ These studies highlight hydrogels' ability to maintain high viral concentrations in target tissues, due to their porous and hydration characteristics. And the controlled virus release is linked to enzymatic processes and the hydrogel's time‐dependent degradation. Lui et al.^165^ have pioneered a transarterial viral embolization technique, encapsulating OA in calcium alginate hydrogel microspheres, enhancing OA therapy's antitumor efficacy for hepatocellular carcinoma and effective in vascular embolization.

In a broader context, hydrogel systems have shown extraordinary prowess in extending the retention time of OVs within tumor tissues, thereby enabling prolonged delivery and ensuring sustained biological activity.^19^ They skillfully impede the nonspecific shedding of OVs to healthy nontarget tissues and deftly evade host immune responses by cloaking viral surface antigens. Ultimately, these innovative hydrogel applications amplify the continuous accumulation, distribution, and lifespan of viruses in tumor tissues, thereby potently catalyzing T‐cell‐mediated antitumor responses and heralding a new dawn in the fight against cancer.

#### 5.2.2 PEG‐related polymer nanoparticles

PEG is a polymer belonging to the polyether class. It is composed of repeating ethylene glycol units, forming a linear polymer structure. Known for its excellent biocompatibility and solubility in water, PEG rarely triggers immune responses, making it an ideal candidate for drug delivery and biomedical materials. Additionally, PEG surfaces can inhibit protein adsorption, reducing nonspecific adsorption when in contact with biological entities. This property helps to maintain the integrity of PEG‐based substrates, preventing protein deposition and cellular adhesion and exhibiting extended blood circulation half‐lives, thus enhancing bioavailability.

PEG polymers play a pivotal role in navigating the complex terrain of humoral immune responses to viruses, significantly boosting viral transduction efficiency. Groundbreaking studies have demonstrated that when PEG is covalently bonded to the surface of adenoviruses, it effectively shields the viral vectors from antibody neutralization.^166^ However, it has been observed that PEGylation of carriers can result in a marked decrease in their ability and efficiency to transduce APCs.^167^ Addressing this challenge, Pau et al. have pioneered the use of poly (β‐amino esters) (pBAEs) as innovative carriers for oncolytic adenoviruses. They ingeniously crafted a stable coating through the electrostatic interaction between positively charged nanoparticles and the negatively charged adenovirus particles. Furthermore, the integration of lipid amine chains, such as hexylamine, into this system fine‐tunes the hydrophilic‐to‐hydrophobic ratio, significantly enhancing cellular affinity and stability toward biological lipid membranes. Remarkably, research indicates that CPEGAd (viral nanoparticles synthesized from pBAEs) can escalate viral transduction rates by a staggering in the milieu of nAbs and effectively thwart the generation of antiadenovirus nAb in vivo^168^ (Figure 5D). Additionally, PEG‐linked maleimide is considered an antigen capture agent. This antigen capture device effectively sequesters tumor‐derived proteins produced by oncolysis, functioning as an in situ vaccine, thereby further triggering an enhanced antitumor adaptive immune response.^169^

#### 5.2.3 Chitosan in polymer nanoparticles

Chitosan, ingeniously derived from the natural polysaccharide chitin through partial deacetylation, emerges as an exemplary material for biocompatible drug delivery systems. Its structural composition, rich in amine groups, can undergo covalent crosslinking with thiol groups, leading to the formation of thiolated chitosan (TC). This modification not only enhances chitosan's stability in polar environments but also amplifies its membrane adhesion capabilities. In the realm of cancer therapy, CD44, a receptor frequently overexpressed on cancer cells and a key player in tumor invasion and migration, is targeted. CD44 acts as a HA‐mediated motility receptor. Thus, the strategic modification of viral particles to target CD44 can significantly heighten tumor specificity, offering substantial advantages in tumor treatment.^170^ Recent breakthroughs in research have showcased that TCs nanoparticles, ingeniously loaded with NDV and functionalized with HA, can effectively achieve active targeting through CD44. This innovative approach also ingeniously shields the virus from the immune system, ensuring an extended release within TME, thereby substantially boosting the virus's bioavailability.^171^

Chitosan also displays remarkable abilities in the activation of macrophages and DCs, triggering cytokine release via interactions with mannose receptors and TLR4. Its inherent cationic nature significantly enhances uptake by APCs, promoting an effective ionic bond with anionic DNA, pivotal for vaccine delivery and antigen presentation. Furthermore, the HA‐coated surface on TCs nanoparticles ingeniously reduces the zeta potential, optimally facilitating the absorption of the MV by anionic cancer cells, particularly in acidic environment. This leads to an outstanding profile of stability and superior controlled‐release characteristics of these nanoparticles.^122^

#### 5.2.4 Polymer nanoparticles for combined therapies

In the cutting‐edge domain of drug delivery, polymer nanocarriers usually integrate with chemotherapeutic agents, forging a path toward enhanced drug release kinetics and precise local drug concentration control in the bloodstream.^172^ A prime example of this innovative synergy is found in cyclodextrin (CD), known for its ability to form complex inclusion compounds with a variety of hydrophobic drugs. The strategic conjugation of CD with the widely used anticancer compound paclitaxel (PTX) leads to the creation of pPTX. This compound distinguishes itself through remarkable water dispersibility and heightened stability.^173^ Expanding on this breakthrough, Kim and colleagues have crafted a sophisticated design of self‐assembling nanoparticles, comprised of pPTX and nitric oxide (NO) infused poly (beta‐CD), referred to as pCD‐pSNO. These nanoparticles stand at the forefront of innovation, displaying significantly augmented cytotoxicity, heightened ICD, robust DCs activation, and amplified T‐cell proliferation. Impressively, the therapeutic impact and benefits of these nanoparticles parallel those observed in OVs.^174^ This pioneering approach not only capitalizes on the individual strengths of each component within the nanocarrier but also creates a powerful synergistic effect, highlighting the immense potential of polymer nanoparticles in the realm of advanced combination therapy techniques.

To date, there have been no reports in the literature of directly using CD as carriers for OVs. However, CD‐based nanocarriers have been extensively studied for the delivery of anticancer drugs, demonstrating their potential in enhancing therapeutic efficacy and safety. This suggests that CD‐based nanocarriers hold promise for future research developments, potentially including applications as carriers for OVs.

Polymer nanoparticles enhance OVT through biocompatibility, targeted delivery, sustained release, and immunomodulatory effects. These advancements pave the way for more precise, efficient, and less toxic treatments, promising significant strides in personalized medicine and theranostics, highlighting the vast potential of polymer nanoparticles in future healthcare applications.

### 5.3 Biologically derived nanocarriers

#### 5.3.1 Cell membrane‐mediated OVs delivery systems

In recent years, the use of biological cell membranes to coat OVs has emerged as a promising approach. This technique confers several unique advantages to OVs, including immune evasion, enhanced targeting specificity, increased stability, and multifunctional modifications. Cell membranes derived from different sources exhibit distinct characteristics, which can be utilized to coat various OVs for the treatment of different types of tumors.

**FIGURE 6:**
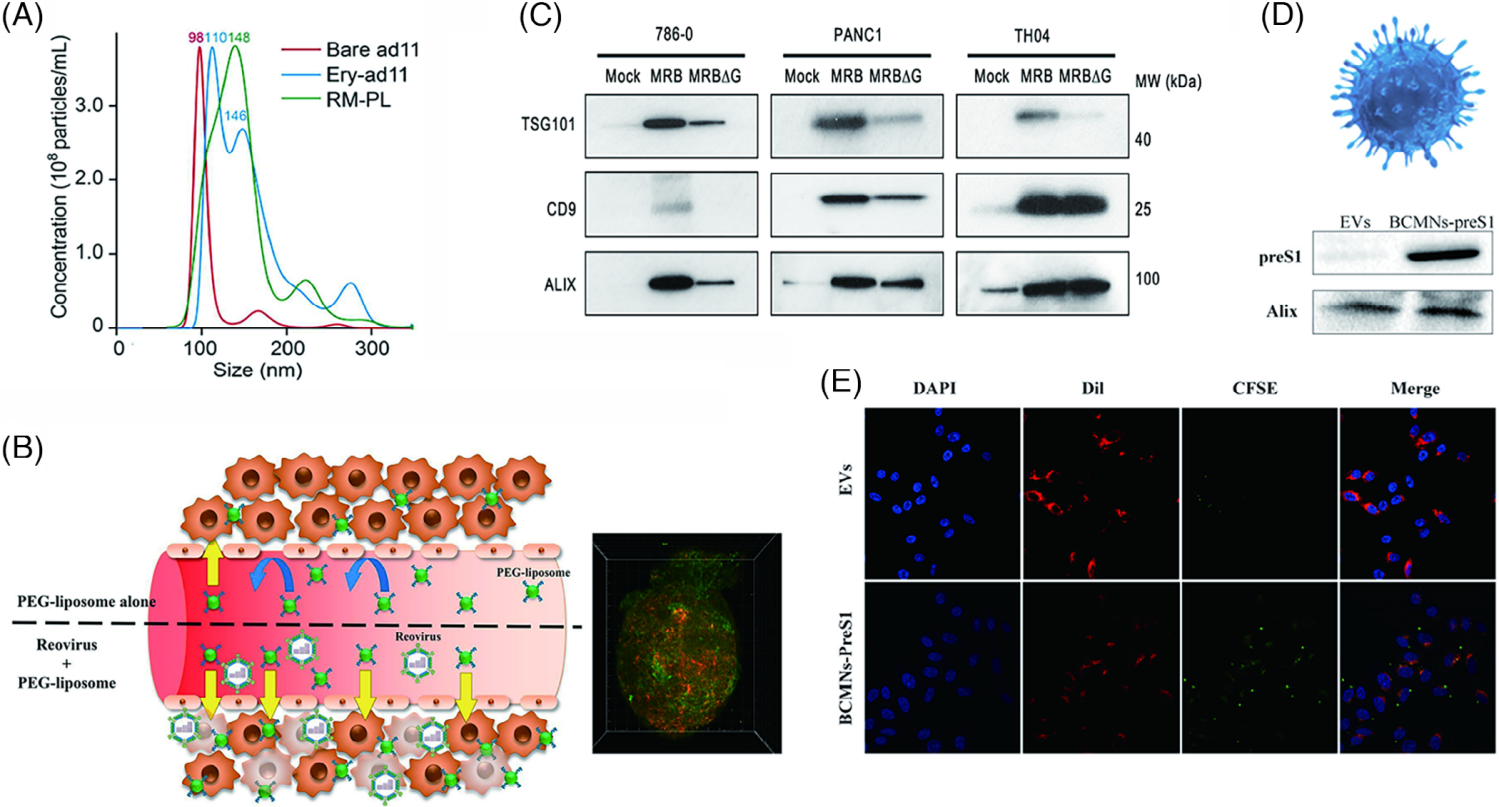
Biologically derived nanocarriers applied to arm OVs. (A) NTA of Ery‐ad11, bare ad11, and red fluorescence signals (RM‐PL): bare ad11 showed a single peak at 98 nm, while RM‐PL showed a peak at 148 nm. In contrast, Ery‐ad11 has one peak each at 146 and 110 nm similar to RM‐PL, demonstrating the successful encapsulation of ad11 with RM‐PL. Reproduced from Ref. 14. Copyright© 2022 Elsevier Ltd. (B) Schematic illustration of pretreatment of intravenous injection of oncolytic reovirus prior to administration of PEG‐liposomes and it showed enhanced PEG‐liposome tumor disposition. Reproduced from Ref. 185. Copyright© 2022 Elsevier B.V. (C) Immunoblotting analysis of total purified secreted EVs from specified cell lines infected with MRB or MRBΔG: Initially, MRBΔG is designed to express only amiR‐4 without being infectious while MRB refers to viruses with normal infectivity. The secreted EVs (TSG101, CD9, and ALIX) by cancer cells is notably increased after oncolytic rhabdovirus infection, a process that may be attributed to the transport of amiR‐4 to uninfected cells via small EVs. Reproduced from Ref. 104. Copyright © 2022, Marie‐Eve Wedge et al. (D) A schematic illustration of BCMNs and Western blot analysis indicating the successful expression of preS1 protein on OA@BCMNs; and (E) confocal laser scanning microscopy (CLSM) images demonstrate that compared with the EVs group, the BCMNs‐PreS1 group exhibits a stronger 5,6‐carboxyfluorescein diacetate succinimidy ester (CFSE) fluorescence signal, suggesting that BCMNs can be surface‐modified with bioactive ligands to acquire specific targeting capabilities. The colors blue, green, and red were used to depict DAPI‐stained cell nuclei, CFSE‐labeled BCMNs‐preS1 and EVs, and sodium taurocholatecotransporting polypeptide (NTCP), respectively, in the visual representation. Reproduced from Ref. 195. Copyright © 2019, American Chemical Society.

Huang et al.^175^ developed an oncolytic VV coated with platelet membranes loaded with indocyanine green (ICG) on the surface. They discovered that this coating significantly increased cellular uptake of the virus complex. Additionally, the platelet membrane enhanced the virus's efficiency in cell invasion and prolonged the retention of ICG induced by the protective properties of the platelet membrane.^175^ Huang's team innovatively fused phospholipids into the red blood cell membrane to form hybrid vesicles. The self‐identifying markers on the red blood cell surface reduced monocyte endocytosis and OV antigen exposure, altering the fluidity and permeability of the liposomes. Through intravenous injection, OV accumulation in tumors increased, enhancing the antitumor efficacy against both primary and metastatic tumors^14^ (Figure 6A). Similarly, NSCs and GBM cells as membrane sources for coating oncolytic adenoviruses has provided a promising OVs‐based targeting strategy for GBM treatment.^112^ Additionally, tumor cells treated with liquid nitrogen can protect OVs from rapid neutralization and elimination. This results in better viral accumulation effects due to the retained surface ligands.^176^ When viruses are encapsulated within the cancer cell membrane, their infectivity and efficacy in vitro are significantly enhanced.^177^Moreover, engineered biomembranes that utilize antigen‐receptor interactions to physically couple oligo‐arginine (OA) to T‐cell specific antigens have demonstrated remarkable OA release capabilities when T‐cells reach tumor cells and recognize homologous tumor‐specific antigens.^178^

Bacterial membranes, due to their unique biological effects, have also endowed OVs with greater therapeutic potential. Bacteria‐derived outer membrane vesicles (OMVs) retain high immunogenicity and leverage pathogenic microorganisms to induce programmed apoptotic necrosis, which is closely related to enhanced antitumor immunity.^179^ Additionally, modifying bacterial OMVs by camouflaging them with biomineralized calcium phosphate (CaP) and engineering them with pyranose oxidase (P_2_O) can reduce clearance by the immune system via the CaP shell and promote P_2_O‐induced ROS production. This process enhances virus‐induced overactive autophagy, leading to cancer cell death.^180^

#### 5.3.2 Lipid nanoparticles

LNPs have been a significant focus in drug delivery research since British scientist Bangham discovered that phospholipids in water can spontaneously form vesicular bilayers, termed liposomes (Bangham and Horne, 1964). These liposomes, resembling the phospholipid bilayer of biological membranes, are often referred to as “artificial biofilms” or “artificial cells.” LNPs are typically composed of four lipid components, including: a phospholipid, cholesterol, a “stealth” lipid, and an ionizable lipid, which are mixed to form homogeneous spheres with 50–150 nm in diameter. The ionizable lipids in LNPs are particularly notable. They carry a charge below physiological pH but become neutral at physiological pH, thus avoiding the inflammatory toxicity often associated with cationic lipids. These lipids both encapsulate the negatively charged nucleic acid payload and are capable of releasing the payload into the cytoplasm at the acidic pH levels present in the endosomal compartment.^181^ Moreover, the unique construction of liposomes enables them to encapsulate both lipophilic substances at the periphery and hydrophilic compounds centrally. This feature allows a wide range of drugs with varying properties to be encapsulated, providing protection from premature clearance by the circulatory and immune systems, inactivation, and dilution.^182^

Liposomes are so capable of encapsulating OVs to shield them from neutralization by antibodies and serving as efficient nanocarriers.^183^ Previously, encapsulation of OVs in liposomes has been shown to modulate the immune response.^184^ Recently, Mendez's team improved adenovirus stability and transfection efficiency in tumor tissues by encapsulating it in anionic lecithin liposomes. This approach overcomes the serum instability issues of cationic liposomes and protects Ad from nAbs, leveraging the negative charge compatibility in physiological environments.^127^ Using standalone OVs in combination with nanoliposome‐encapsulated OVs as a synergistic oncolytic therapy represents an innovative approach. In one experiment, a pretreatment with Coxsackievirus was administered to a B16 mouse model to induce apoptosis in tumor cells. This was followed by the application of PEGylated liposomes. After 16 days of treatment, a significant improvement in tumor management in the mice was observed^185^ (Figure 6B). Tumor neovascularization plays a crucial role in tumor growth, infiltration, and metastasis. The peptide iRGD specifically binds to integrin αvβ3 receptors, which are highly expressed in the tumor neovascular system, thereby blocking intratumoral angiogenesis. Endothelium‐targeted iRGD liposomes encapsulating recombinant NDV expressing the DCs‐related chemokine, macrophage inflammatory protein‐3α (MIP‐3α), were found to inhibit tumor angiogenesis and reverse the immunosuppressive TME in B16 and 4T1 mice.^186^ Another significant challenge is how to increase the accumulation of OVs at the tumor site. Capitalizing on the tendency of parthenogenic anaerobic bacteria to colonize TME, researchers have bioconjugated liposome‐encapsulated OVs with tumor‐homing Escherichia coli. This ‘bacteria‐OV coupling’ strategy successfully enhanced OV enrichment at tumor sites, boosting antitumor immunity.^103^

#### 5.3.3 Extracellular vesicles

EVs are nanometer‐sized lipid bilayer vesicles of cellular origin with good biocompatibility, low immunogenicity and low toxicity, they are considered ideal nanocarriers for drug delivery. The protein phenotype of EVs has emerged as a promising biomarker for cancer diagnosis and therapeutic monitoring.^187^ Particularly, cancer‐derived EVs exhibit selective tropism for tumor tissues of vesicular origin and even some heterologous, cross‐species tumor predisposition.^188^ In fact, cancer is the acquisition of some malignant counterparts or pathways by tumor cells to promote their own proliferation after exchanging information through corresponding hormones, proteins, metabolites, and extracellular vesicular EVs.^189^ Pathogenic viruses can also transfer virally encoded gene products via EV to neighboring uninfected cells to enhance viral growth in normal tissues.^190^ Expanding upon these discoveries, Wedge et al.^104^ conducted a screening for an artificial microRNA encoded by the virus, named amiR‐4, which is designed to target some key proteins in chromatin remodeling that significantly contributes to the resistance against OV replication. Their findings demonstrated that amiR‐4, when expressed by the virus, is distributed among cells through small EVs, enabling its transfer to cells not infected by the virus. This mechanism facilitates the bystander elimination of adjacent, uninfected cancer cells, substantially amplifying the cytotoxic impact on tumor cells^104^ (Figure 6C). Furthermore, the telomerase‐specific oncolytic adenovirus, telomolysin (OBP‐301), demonstrated a distal effect following local treatment in immunodeficient mice. This effect is hypothesized to result from the transport of materials to distal tumors via tumor cell‐derived EVs.^191^ Research has also shown that EVs released from cells infected with oncolytic adenovirus can reinfect other cells. These infected EVs (IEVs) carry sufficient viral DNA and proteins to enhance viral replication in vitro.^192^ These findings underscore the significant potential of EVs in the realm of drug delivery for anticancer therapy.

EVs can also be used in combination with conventional chemotherapy. In a study investigating the combined treatment of oncolytic HSV and cisplatin in platinum‐resistant ovarian cancer cells, researchers observed disruption in EVs‐related pathways within infected cells. This disruption led to a reduction in the expression of transport proteins on EVs associated with cisplatin efflux, resulting in increased intracellular retention of cisplatin. As EVs can directly mediate the efflux of drugs within tumor cells, this demonstrated an enhanced intratumoral DNA damage and activation of inflammatory signaling pathways.^193^ A research team has conducted systemic delivery of oncolytic adenovirus and PTX encapsulated within EVs, demonstrating higher transduction efficiency and enhanced infectious titer compared with the separate use of the virus and PTX. Notably, encapsulating PTX in EVs effectively induces beneficial immune responses in the tumor vicinity without altering the OVs' capacity to stimulate tumor‐associated inflammatory reactions, including NF‐κB activation, immunogenicity, and CD3^+^, CD4^+^, and CD8^+^ T‐cell infiltration. This approach mitigates the systemic inflammatory responses typically associated with conventional PTX therapy.^123^, ^194^

EVs have been genetically engineered for enhanced antiviral shielding and targeted cancer cell specificity. For instance, this approach includes two methods: in vitro genetic membrane engineering and in vivo CRISPR expression of targeting ligands on red blood cell membranes. The targeting peptide preS1 is integrated into nanovesicles, boosting the targeting delivery of EVs. Encapsulating oncolytic adenovirus in these bioengineered cell membrane nanovesicles (BCMNs) preserves its infectivity, facilitates evasion from innate immune responses, and effectively targets tumor sites. BCMNs blend natural cell membrane functionalities with bioengineered targeting, contributing to advancements in cancer virotherapy^195^ (Figure 6D,E).

## 6 NANOTECHNOLOGY ARMED WITH ONCOLYTIC VIRUSES

### 6.1 Photodynamic therapy

PDT is an oncology therapy with greater potential due to its spatiotemporal specificity and having the advantage of being noninvasive. PDT inhibits tumor growth by activating the generation of singlet oxygen from photosensitizers (PSs) based on a specific laser light, causing an oxidative reaction of adjacent biomolecules, resulting in a cytotoxic effect.^196^ Furthermore, the pathogen‐associated molecular patterns and DAMPs released from the destroyed cancer cells recruit and activate immune cells (macrophages, neutrophils, and DCs), release additional proinflammatory cytokines (TNF‐α and IL‐1β), decrease anti‐inflammatory cytokines (IL‐10), improve TME, and further enhance immune cell infiltration.^197^, ^198^ However, there are many challenges to PDT‐mediated ICD induction, such as the short half‐life and aggregation‐induced burst effect of most PSs, the dependence of ROS generation by PDT on the sparse oxygen supply in solid tumors, and the difficult to ameliorate immunosuppressive effects in TME.^199^ The combined use of nanomedical technologies with PDT is expected to provide a possible solution to the bottlenecks of existing PDTs. These include: improving the physicochemical properties and half‐life of PSs, enhancing the targeted aggregation effect, prolonging the blood circulation time, and remodeling the hypoxic TME.

**FIGURE 7:**
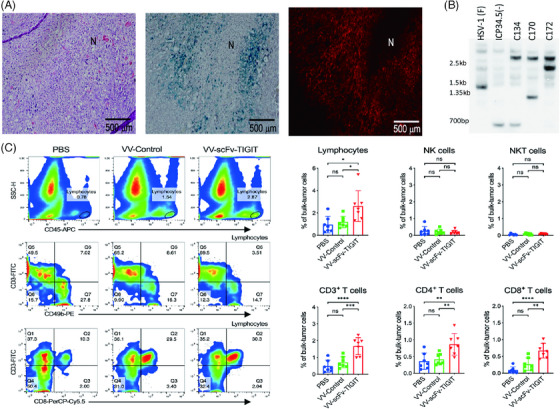
The integration of nanotechnologies and OVs. (A) Left, hematoxylin and eosin staining of human MM (IOMM‐Lee) tumors postintratumoral injection of G47Δ‐KR; middle, X‐gal staining revealing the presence and distribution of G47Δ‐KR infection within the IOMM‐Lee tumors; right, fluorescence microscopy detecting KillerRed (KR) expression in the necrotic regions of the IOMM‐Lee meningioma. In summary, the photosensitive protein KR is successfully expressed in the IOMM‐Lee meningioma model. N refers to necrotic area at the center where the virus was inoculated. Reproduced from Ref. 202. Copyright © 2023 Elsevier B.V. (B) Southern blot results for the recombinant strains: C170 exhibits bands at 1.2 and 3.0 kb, while C172 shows bands at 2.3 and 3.0 kb. Both display the expected *Nco*I fragments, confirming the successful expression of EphA2 on the viral surface. Reproduced from Ref. 205. Copyright © 2021, BMJ Publishing Group Ltd & Society for Immunotherapy of Cancer. (C) Flow cytometry analysis showed that intratumoral injection of VV‐scFv‐TIGIT significantly increased the infiltration of CD45^+^lymphocytes, CD3^+^T, CD4^+^T, and CD8^+^T cells. Reproduced from Ref. 212. Copyright © 2021, BMJ Publishing Group Ltd & Society for Immunotherapy of Cancer.

There have been several reports combining PDT with OV therapy, demonstrating better tumor therapeutic effects. Engineered OV can enhance the antitumor efficacy of OV by manipulating VEGF‐mediated signaling.^200^ In their study, Gil et al.^201^ initially subjected a mouse tumor model to PDT, resulting in subtle damage to the vascular endothelium. Twelve hours later, oncolytic poxvirus was intravenously injected. The PDT‐induced damage to the vascular endothelial gaps and dysfunction facilitated the extravasation of virus particles, promoting virus infection.^201^ It was also possible to genetically engineer the clinical tumorolytic HSV G47Δ expressing the photosensitive protein KillerRed (KR), which produced higher ROS as well as cell death in an in vitro model of human GBM and malignant meningioma^202^ (Figure 7A). High drug accumulation at the tumor site is one of the key aspects of the OV method. The chemical conjugation of MNPs with adenovirus enhances the virus's infectivity. Additionally, the appropriate strength of the magnetic field, through microscopic “point” manipulation, facilitates remote guidance from the organ and tissue level to the microscopic level. This results in superior tumor targeting and particle accumulation. This promotes the concentrated expression of the photosensitive protein KR within cells, achieving sensitization. Ultimately, this results in highly specific light‐triggered virotherapy.^203^ Furthermore, the application of PDT has been found to effectively induce ICD and enhance immune responses. Therefore, the therapeutic effectiveness of PDT may involve the remodeling of tumor tissue and immune responses, warranting further in‐depth investigation.^204^

### 6.2 Genetic engineering

Genetic engineering, a sophisticated branch of genetic technology, involves constructing hybrid DNA molecules from diverse sources based on a meticulously designed in vitro blueprint. These custom‐engineered molecules are then introduced into living cells, enabling the modification of inherent genetic traits, the creation of novel varieties, and the production of groundbreaking products. With the relentless progression of biotechnological methods, the field of cancer therapy has witnessed a quantum leap through the development of genetically modified or reengineered OVs, positioning them at the forefront of cancer immunotherapy research.

In the realm of OVs, a plethora of scientists employ genetic engineering to refine viruses to improve the effect of antitumor immunity. They are broadly divided into the following two ideas: (a) to modify the target recognition receptors and cell death related receptors/ligands on the surface of viruses to achieve better target recognition and tumor cell death; (b) to promote the expression of proinflammatory cytokines, chemokines and immune checkpoint‐related antibodies after viral infection of cells to improve the inhibitory state of the TME.

Illustrative of these advancements, a multimodal oncolytic HSV, expressing the shared TAA ephrin A2 (EphA2), elicits a potent acquired antitumor immune response in oncolytic HSV‐resistant murine brain and peripheral tumor models. Furthermore, it gives rise to a functional population displaying an augmented Epha2 antigen response, effectively suppressing tumor growth upon rechallenge in survivor mice^205^ (Figure 7B). The oncolytic adenovirus rAd5pz‐zTRAIL‐RFP‐SΔ24E1a (A4), which carries the TNF‐related apoptosis‐inducing ligand (TRAIL) associated with the viral capsid protein IX, demonstrates enhanced therapeutic potential. To further optimize A4, the research team developed a new iteration, zA4, by coating additional soluble TRAIL onto A4. This modification results in increased infection of cancer cells and improved targeting of tumors.^206^, ^207^ Additionally, a recombinant oncolytic NDV, capable of expressing the DCs‐specific chemokine, namely MIP‐3α, is employed to augment tumor lysis and facilitate systemic antitumor immunity.^208^ Adenoviruses encoding IL‐2 and TNF‐α yield remarkable outcomes in experiments, including enhanced tumor control, elevated intratumoral Th1/Th6 cytokine levels, and increased infiltration of CD1^+^ T cells and CD8^+^ DCs.^209^

As mentioned above, in addition to direct tumor lysis, OVs induce antitumor immunity by recruiting and activating immune cells in the local TME. However, this activation is often hindered by an inhibitory TME, especially due to the elevated expression of immune checkpoints in cells, potentially diminishing OV effectiveness.^210^ To address this challenge, an influenza A virus (IAV) expressing a single‐chain antibody targeting the immune checkpoint CTLA4 (IAV‐CTLA4) has been designed. In experiments, this modified virus demonstrated slowed tumor growth, mediated systemic effects, and improved overall survival rates.^211^ Moreover, TIGIT, an emergent member of the immunoglobulin superfamily and a coinhibitory receptor regulating NK cells and T cells activation, has been targeted. Zuo et al. engineered VV–scFv–TIGIT through homologous recombination with parental VV. This construct, encoding a single‐chain variable fragment (scFv) targeting the T cell immunoglobulin and ITIM domain (TIGIT), has shown remarkable tumor‐curing effects in a murine model of malignant ascites, enhancing T cell infiltration and CD8^+^T cell activation^212^ (Figure 7C).

### 6.3 Technologies related to real‐time viral monitoring

The prerequisites for the clinical use of OVs in tumor therapy are reliable and rapid detection and quantitative analysis. Currently, researchers have developed various methods to detect virus‐related properties both in vivo and in vitro, primarily based on the fluorescence and magnetic properties of these viral particles.

A critical aspect in the production of viral nanoparticles is their detailed characterization.^213^ Nanoparticle tracking analysis (NTA) is a sophisticated technology employed for tracking and monitoring nanoparticles. This technique is instrumental in determining the size, dimensions, zeta potential, and aggregation states of viruses during production. Virus particles are labeled with fluorescent dyes and irradiated with a laser. These particles are then observed using a high‐sensitivity metal‐oxide‐semiconductor camera connected to an optical microscope, enabling real‐time analysis of virus aggregation and dissolution kinetics.^214^ Similarly, the helium ion microscope (HIM) is a powerful imaging tool that uses a helium ion beam for high‐resolution imaging. With its high resolution, sensitivity, nondestructive nature, and 3D imaging capabilities, HIM is well‐suited for characterizing OVs within encapsulating materials.^194^, ^215^

In addition, a genetically encoded turn‐on fluorescent biosensor, cVisensor, the cyclized viral sensor, consists of structurally distorted green fluorescent protein (GFP) by protein cyclization facilitated by the potent Nostoc punctiforme DnaE intein (Npu DnaE). Adenoviral protease (AVP) is closely associated with viral polyprotein processing and precapsid maturation, and the addition of AVP‐cleavable sequences to the above sensors mitigates distortion and subsequent fluorescence emission. HEK‐293 sensor cells, stably expressing cVisensor were used for live‐cell monitoring of adenoviral infections and to establish a reproducible flow cytometry‐based bioassay for reliable label‐free quantification of infectious adenoviruses at 2 days postinfection. It is faster compared with flow cytometry and can reduce the time and cost of AdV preparation, titration and characterization.^216^

## 7 CONCLUSIONS AND OUTLOOK

In the past few decades, the application of OVs has undergone rapid development. From the early recognition over a century ago that OVs could kill cancer cells, to recent years where numerous clinical trials have validated the oncolytic efficacy of viruses benefiting the survival of cancer patients, to the current era of genetically engineering viruses and rearming them with different nanomaterials for immunotherapeutic applications—such armed OVs represent a promising frontier. Encouragingly, several OV drugs have obtained regulatory approval for clinical trials, such as talimogene laherparepvec (T‐VEC;Imlygic), Oncorine (H101), and Delytact (Teserpaturev). Notably, Imlygic, a genetically modified HSV‐1 developed by Amgen, was the first OVT approved by the FDA for the treatment of melanoma in 2015. Delytact, the latest OV based on HSV‐1, is the first approved for the treatment of malignant gliomas. H101, a recombinant oncolytic adenovirus, can be combined with chemotherapy to treat nasopharyngeal cancer. However, due to various limitations of OVs, their monotherapy efficacy falls short of the ideals, and currently, their applications lag behind mature drugs like immune checkpoint inhibitors (ICIs).

Recent breakthroughs and innovative applications of various technologies provide new opportunities for the further development of OVs. This review aims to provide unique insights into the development of OVs, offering guidance to new researchers for a quick understanding of the field and its cutting‐edge breakthroughs. It also addresses the rational design of OVs armed with different nanotechnologies, striving for greater progress in the field of clinical translation. The intricate interplay between OVs and the complex tumor immune mechanisms necessitates more experiments to decipher and grasp the delicate balance, applying various technologies to empower the viruses. In this process, numerous challenges and questions inevitably arise.

(A) The production of OVs

The prerequisite for the practical clinical application of mature OVs lies in perfecting the production technology, particularly in characterizing the virus particles coated with different materials. Currently, most experiments are limited to in vitro settings, involving the small‐scale production of viruses through infecting cells and synthesizing virus‐material complexes. Large‐scale production capabilities are yet to be developed. Furthermore, there is a lack of technology for real‐time monitoring and quality control of the synthesis of virus‐material particles. Future research should focus on optimizing virus production technology.

(B) The administration of OVs

(A) The various administration routes of oncolytic vaccines include subcutaneous, intradermal, intramuscular injection, and intralymph node injection, and each has its benefits and challenges for different vaccines. The choice of administration route affects the biological distribution, antigen presentation, and subsequent immune responses of delivered vaccines. Ascertaining the optimal dosage and timing of personalized vaccines for each route is crucial for maximizing their efficacy while minimizing potential adverse reactions. (B) The intercellular connections within epithelial cells and ECM serve as barriers to therapeutic penetration in tumor tissues, potentially leading to drug resistance. In current experiments, the barrier effect of tumors has been somewhat diminished due to the use of intratumoral injections. Additionally, the induction of apoptosis and caspase‐8 activation by cytotoxic drugs leads to increased infiltration within tumors. This may be attributed to the contraction or removal of apoptotic cells, generating channel‐like structures and pores that facilitate the spread of oncolytic HSV.^217^ Exploring ways to simulate the authentic tumor environment and assess the therapeutic efficacy of OVs in cancer remains an ongoing exploration.

(C) The application of OVs

The identification of suitable patient populations for OVT remains a subject of ongoing research. In clinical practice, OVs are not typically the first‐choice treatment, and not all patients' tumors harbor identifiable new antigens. Due to the experimental nature of most OVs, patients undergoing OVT often have undergone multiple rounds of various conventional or unconventional cancer treatments.

This particular patient group often exhibits compromised immune systems, posing a question mark on the effectiveness of OVs. The altered immune microenvironment resulting from previous treatments may deviate significantly from the original design intent. Reliable immune markers or any other type of predictive indicators are essential to be further explored. This exploration aims to better match the individual differences among cancer patients and achieve enhanced effectiveness in tumor treatment.

(D) Monitoring and evaluation

The majority of research related to OVs is predominantly confined to in vitro settings, resulting in an insufficient understanding of the effective and safe viral loads within the body. Clinical assessment requires the tracking of objective responses, such as tumor size reduction, progression‐free survival, and overall survival. Simultaneously, consideration of the patient's quality of life is crucial. More in vivo research is needed to identify biomarkers that can demonstrate the topographical location and functional state changes of the virus within the organism, predicting the efficacy of OVT. Bernstock and colleagues have established a multibiomarker screening platform for evaluating the response of pediatric GBM to viral therapy.^218^ Effective dosage selection must meet the requirements of the TME. The delicate balance between mediating antiviral and antitumor immune responses is also of paramount importance. Furthermore, while OVT can effectively limit the initial treatment response in tumors, careful construction of multiarm or systemic delivery vectors is necessary. This ensures tumor‐specific expression and prevents systemic toxicity.

(E) Personalized cancer vaccines

The integration of oncolytic therapy with other treatment modalities provides a novel approach in the field of cancer treatment. By combining different therapeutic approaches, there is the potential to enhance treatment efficacy, overcome drug resistance mechanisms, and ultimately improve patient prognosis. Recent scientific discoveries emphasize the potential synergistic effects of combining oncolytic vaccines with other forms of immunotherapy. Notably, ICIs targeting PD‐1 (programmed cell death protein 1), PD‐L1 (programmed death‐ligand 1), and CTLA‐4 (cytotoxic T‐lymphocyte‐associated protein 4) in conjunction with personalized cancer vaccines has shown promising results. In addition to traditional and well‐known cancer treatments such as radiotherapy, chemotherapy, and immunotherapy (mostly ICIs), oncolytic therapy can be effectively combined with other innovative nanotechnologies. For instance, NanoKnife, a nonthermal ablation technique also known as irreversible electroporation, has been employed in the treatment of locally advanced pancreatic cancer. In a study, the combination of NanoKnife with oncolytic adenovirus M1 in an “electrochemotherapy” approach induced by NanoKnife‐induced electroporation provided nonreceptor‐dependent membrane channels for M1 virus. This facilitated virus infection and triggered ROS‐dependent apoptosis in PCCs mediated by the inhibition of the phosphoinositide 3‐kinase/protein kinase B pathway. In vitro experiments significantly extended the survival period of the in situ immune‐active mouse PC model.^219^ Therefore, there is encouragement for the collaborative use of OVs with a broader range of nanotechnologies to seek improved biosafety and therapeutic outcomes in cancer treatment.

## AUTHOR CONTRIBUTIONS

Y. Z. and K. Z. proposed this theme, organized the skeleton, provided the raw materials, and selected the cited references. Y. Z., X. S., G. C., Y. S., X. D., R. H., Y. Z., and H. Tan reorganized figures and wrote the manuscript. K. Z. revised this manuscript, supervised and supported the project. All authors commented on this manuscript.

## CONFLICT OF INTEREST STATEMENT

The authors declare no conflict of financial interest.

## Data Availability

Not applicable.
